# A KSHV microRNA Directly Targets G Protein-Coupled Receptor Kinase 2 to Promote the Migration and Invasion of Endothelial Cells by Inducing CXCR2 and Activating AKT Signaling

**DOI:** 10.1371/journal.ppat.1005171

**Published:** 2015-09-24

**Authors:** Minmin Hu, Cong Wang, Wan Li, Weiping Lu, Zhiqiang Bai, Di Qin, Qin Yan, Jianzhong Zhu, Brian J. Krueger, Rolf Renne, Shou-Jiang Gao, Chun Lu

**Affiliations:** 1 State Key Laboratory of Reproductive Medicine, Nanjing Medical University, Nanjing, P. R. China; 2 Key Laboratory Of Pathogen Biology Of Jiangsu Province, Nanjing Medical University, Nanjing, P. R. China; 3 Department of Microbiology, Nanjing Medical University, Nanjing, P. R. China; 4 Department of Pathology, the First Affiliated Hospital of Nanjing Medical University, Nanjing, P. R. China; 5 Department of Endocrinology and Metabolism, Huai’an First People’s Hospital, Nanjing Medical University, 6 Beijing Road West, Huai’an, Jiangsu, P. R. China; 6 Cancer Virology Program, University of Pittsburgh Cancer Institute, Pittsburgh, Pennsylvania, United States of America; 7 Department of Molecular Genetics and Microbiology, University of Florida, Gainesville, Florida, United States of America; 8 Department of Molecular Microbiology and Immunology, Keck School of Medicine, University of Southern California, Los Angeles, California, United States of America; University of North Carolina at Chapel Hill, UNITED STATES

## Abstract

Kaposi's sarcoma (KS) is a highly disseminated angiogenic tumor of endothelial cells linked to infection by Kaposi's sarcoma-associated herpesvirus (KSHV). KSHV encodes more than two dozens of miRNAs but their roles in KSHV-induced tumor dissemination and metastasis remain unknown. Here, we found that ectopic expression of miR-K12-3 (miR-K3) promoted endothelial cell migration and invasion. Bioinformatics and luciferase reporter analyses showed that miR-K3 directly targeted G protein-coupled receptor (GPCR) kinase 2 (GRK2, official gene symbol *ADRBK1*). Importantly, overexpression of GRK2 reversed miR-K3 induction of cell migration and invasion. Furthermore, the chemokine receptor CXCR2, which was negatively regulated by GRK2, was upregulated in miR-K3-transduced endothelial cells. Knock down of CXCR2 abolished miR-K3-induced cell migration and invasion. Moreover, miR-K3 downregulation of GRK2 relieved its direct inhibitory effect on AKT. Both CXCR2 induction and the release of AKT from GRK2 were required for miR-K3 maximum activation of AKT and induction of cell migration and invasion. Finally, deletion of miR-K3 from the KSHV genome abrogated its effect on the GRK2/CXCR2/AKT pathway and KSHV-induced migration and invasion. Our data provide the first-line evidence that, by repressing GRK2, miR-K3 facilitates cell migration and invasion via activation of CXCR2/AKT signaling, which likely contribute to the dissemination of KSHV-induced tumors.

## Author Summary

Kaposi's sarcoma-associated herpesvirus (KSHV) is the etiological agent of Kaposi's sarcoma (KS). KS is a highly disseminated tumor often involved with visceral organs. Experimentally, KSHV infection induces the invasiveness of endothelial cells. KSHV encodes twelve precursor miRNAs (pre-miRNAs), which are processed into at least 25 mature miRNAs. However, the roles of these miRNAs in KSHV-induced tumor dissemination remain unknown. Here, we investigated KSHV-encoded miR-K12-3 (miR-K3) promotion of endothelial cell migration and invasion, which are the underlying mechanisms of tumor dissemination. We demonstrated that miR-K3 promoted cell migration and invasion by directly targeting G protein-coupled receptor (GPCR) kinase 2 (GRK2). Furthermore, we found that the chemokine receptor CXCR2, which was negatively regulated by GRK2, and its downstream AKT signaling positively mediated miR-K3-induced cell migration and invasion. miR-K3 downregulation of GRK2 relieved its direct inhibitory effect on AKT, and both CXCR2 induction and the release of AKT from GRK2 were required for miR-K3 maximum activation of AKT and induction of cell migration and invasion. These results show that miR-K3 and its the downstream pathway may be potential therapeutic targets for the treatment of KSHV-associated malignancies.

## Introduction

Kaposi’s sarcoma-associated herpesvirus (KSHV) is a gammaherpesvirus associated with Kaposi’s sarcoma (KS) commonly seen in AIDS patients [1]. KSHV is also linked to other lymphoproliferative diseases including primary effusion lymphoma (PEL) and multicentric Castleman’s disease (MCD) [1–3]. As an angioproliferative malignancy of human skin derived from endothelial cell lineage, KS is histologically characterized by abnormal and leaky vessels, extravasated erythrocytes with hemosiderin deposits, and vast inflammatory infiltration [4]. The early manifestations of KS usually appear in skin and lymph nodes; however, advanced KS often behaves as a highly disseminated tumor involved with visceral organs including the respiratory and gastrointestinal tract [5,6]. Among the four distinct clinical variants of KS (classic KS, endemic KS, iatrogenic KS and AIDS-KS), AIDS-KS is the most common and aggressive form and frequently occurs throughout the body, including skin of the face, torso, mucous membranes of the oral cavity, the respiratory tract, lungs and intestines [7]. Notably, distal dissemination or metastasis is often observed in KS and AIDS-KS patients and causes diffuse lung disease, such as pulmonary KS. Therefore, understanding of the molecular basis underlying KS tumor dissemination could shed lights on the mechanism of AIDS-KS pathogenesis and lead to the development of rational therapies.

MicroRNAs (miRNAs) are 19–23 nucleotides-long small noncoding single-stranded RNAs that act post-transcriptionally to regulate the expression of large numbers of genes in eukaryotic genomes by targeting the complementary gene sequences through its seed region. Typically, miRNAs bind to complementary sequences within the 3’ untranslated region (UTR) of a target gene leading to mRNA degradation or down-regulation of translation [8]. KSHV encodes 12 precursor miRNAs (pre-miRNAs) within the latency-associated region, which are processed into at least 25 mature miRNAs named as KSHV-miR-K12-1~12 (or simply as miR-K1~12) [9–11]. KSHV miRNAs are highly expressed during latency and in KS tumors [9,10,12–14], implying their essential functions in the viral life cycle and the development of KS tumors. Indeed, several KSHV miRNAs have been reported to regulate viral latency by directly targeting viral genes or indirectly targeting cellular pathways [15–21]. For instance, miR-K5, miR-K7-5p, miR-K9-5p target KSHV lytic switch protein (RTA) and regulate KSHV latency [15,17,19]. In addition, miR-K3 has been found to target nuclear factor I/B (NFIB) and indirectly inhibit RTA to regulate the viral life cycle [20]. A recent study has indicated that miR-K11 and miR-K3 contribute to the maintenance of latency by decreasing RTA expression via down-regulation of MYB, C/EBPα and Ets-1 [22]. KSHV miRNAs also regulate apoptosis, cell cycle, cytokine production and secretion, immune evasion, angiogenesis, epigenetics and cellular transformation by directly regulating KSHV and/or host genes [16,23–39], which might contribute to the development of KSHV-related malignancies [40]. However, whether KSHV-encoded miRNAs participate in the dissemination or metastasis of the KS tumors, which reflected in the migratory and invasive abilities of the tumor cells, is still uncertain.

Because of the high expression levels of miR-K3 in KS lesions [41], in this study, we examined the miR-K3 regulation of endothelial cell migration and invasion. We have found that in addition to NFIB, C/EBPα and Ets-1, miR-K3 directly targeted G protein-coupled receptor kinase 2 (GRK2, official gene symbol *ADRBK1*), which has initially been identified as a serine/threonine kinase implicated in the regulation of multiple G protein-coupled receptors (GPCRs) with arrestins and modulation of cell motility by the complex [42]. Furthermore, we have identified CXCR2/AKT signaling axis, which is negatively regulated by GRK2, mediates miR-K3-induced endothelial cells migration and invasion. This is the first report to describe the involvement of a KSHV-encoded miRNA in endothelial cell migration and invasion. As the deregulation of cell migration and invasion during KS progression determines the capacity of tumor cells to escape from the primary tumors and invade adjacent tissues, our findings reveal a novel mechanism by which KSHV miRNAs contribute to the pathogenesis of KSHV-related malignancies.

## Results

### Ectopic Expression of MiR-K3 Promotes Migration and Invasion of Endothelial Cells

**Fig 1:**
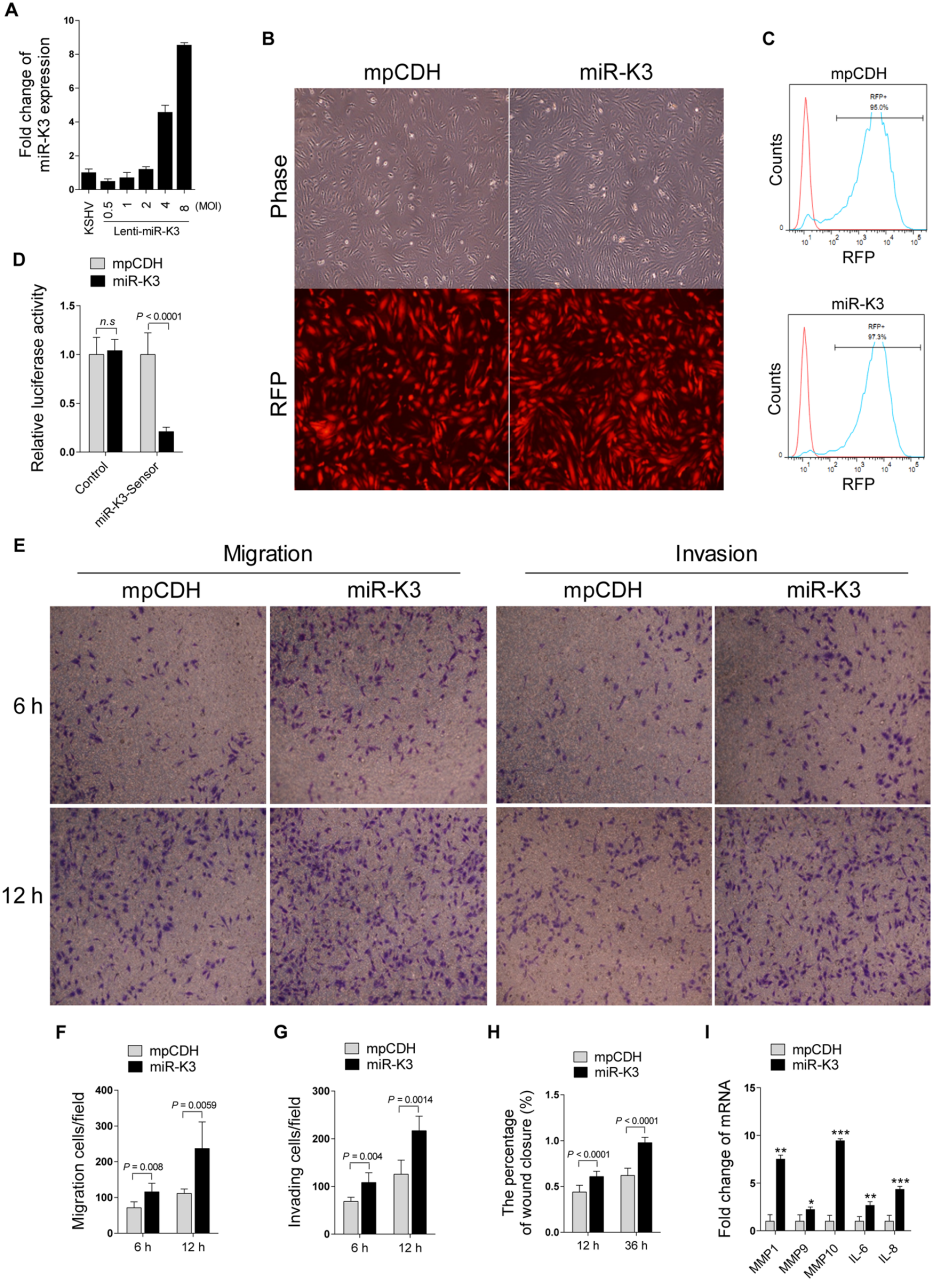
Ectopic expression of miR-K3 promotes endothelial cell migration and invasion. **(A)**. KSHV miR-K3 expression in HUVEC infected with KSHV BAC16 virus induced from iSLK-BAC16 cells or transduced by the different MOI of lentiviral miR-K3 were determined by qPCR. The miR-K3 level in KSHV group was set as ‘‘1” for comparison. **(B)**. HUVEC were transduced with 2 MOI of lentivirus empty vector (**mpCDH**; left) and lentivirus-miR-K3 (**miR-K3**; right), and representative images were taken under light microscope (**Phase**; top) and fluorescent microscope (**RFP**; bottom) (Original magnification, ×100). **(C)**. Cells treated as in (B) were analyzed for RFP expression by flow cytometry to determine transduction efficiency. *y* axis units are numbers of cells. **(D)**. Luciferase activity was detected in 2 MOI of lentivirus empty vector (**mpCDH**) or lentivirus-miR-K3 (**miR-K3**) transduced HUVEC transfected by the pGL3-Control (**Control**) or the pGL3-miR-K3 sensor reporter (**miR-K3-Sensor**). *** *P* < 0.001 for Student’s *t*-test. *n*.*s*., not significant. **(E)**. Transwell migration (left panel) and Matrigel invasion (right panel) assays for HUVEC transduced with lentivirus empty vector (**mpCDH**) or lentivirus-miR-K3 (**miR-K3**). The representative images were captured at 6 and 12 h post seeding (original magnification, ×100). **(F)**. The quantification results of Transwell migration assay in (**E**). **(G)**. The quantification results of Matrigel invasion assay in (**E**). **(H)**. The quantification results of wound healing assay in S1 Fig. **(I)**. The mRNA expression of MMP1, 9, 10 and IL-6, 8 in HUVEC treated as in (**B**) were determined by qPCR.

It has been reported that KSHV infection facilitates the migration and invasion of endothelial cells, and several KSHV-encoded genes and cellular miRNAs have been shown to participate in this process [6,43,44]. To determine whether KSHV-encoded miRNAs were also involved in regulating the migration and invasion of endothelial cells, HUVEC were transduced with the different MOI of lentivirus expressing miR-K3. At 2 MOI, miR-K3-transduced HUVEC exhibited a miR-K3 expression level similar to that of KSHV (BAC16)-infected HUVEC (Fig 1A). Thus, we chose 2 MOI for the following transduction experiments. Under this condition, over 95% cells were RFP-positive at day 3 or 4 post-transduction, indicating the successful lentivirus transduction (Fig 1B and 1C). Expectedly, miR-K3 markedly inhibited the reporter activity of pGL3-miR-K3 sensor reporter, indicating that the miR-K3 expression construct in HUVEC was functional (Fig 1D). In Transwell migration and Matrigel invasion assays, HUVEC transduced with miR-K3 exhibited increased levels of migration and invasion when compared with those transduced with the vector control (Fig 1E, 1F and 1G). Consistently, wound healing assays showed that HUVEC transduced with miR-K3 had increased level of motility compared to those transduced with the vector control (Fig 1H and S1 Fig). Besides migration and invasion, we also have screened many other phenotypes including cell proliferation, cell cycle, and plate colony formation, however, we found that miR-K3 did not affect these phenotypes’ changes. Furthermore, qPCR was performed to detect several cytokines that are related to cell migration and invasion. We found that miR-K3 upregulated the levels of transcripts of matrix metalloproteinases (MMPs) 1, 9 and 10, and inflammatory cytokines IL-6 and IL-8 by 2–9 folds compared to the control vector (Fig 1I).

### GRK2 is Down-Regulated by KSHV and Directly Targeted by MiR-K3

**Fig 2:**
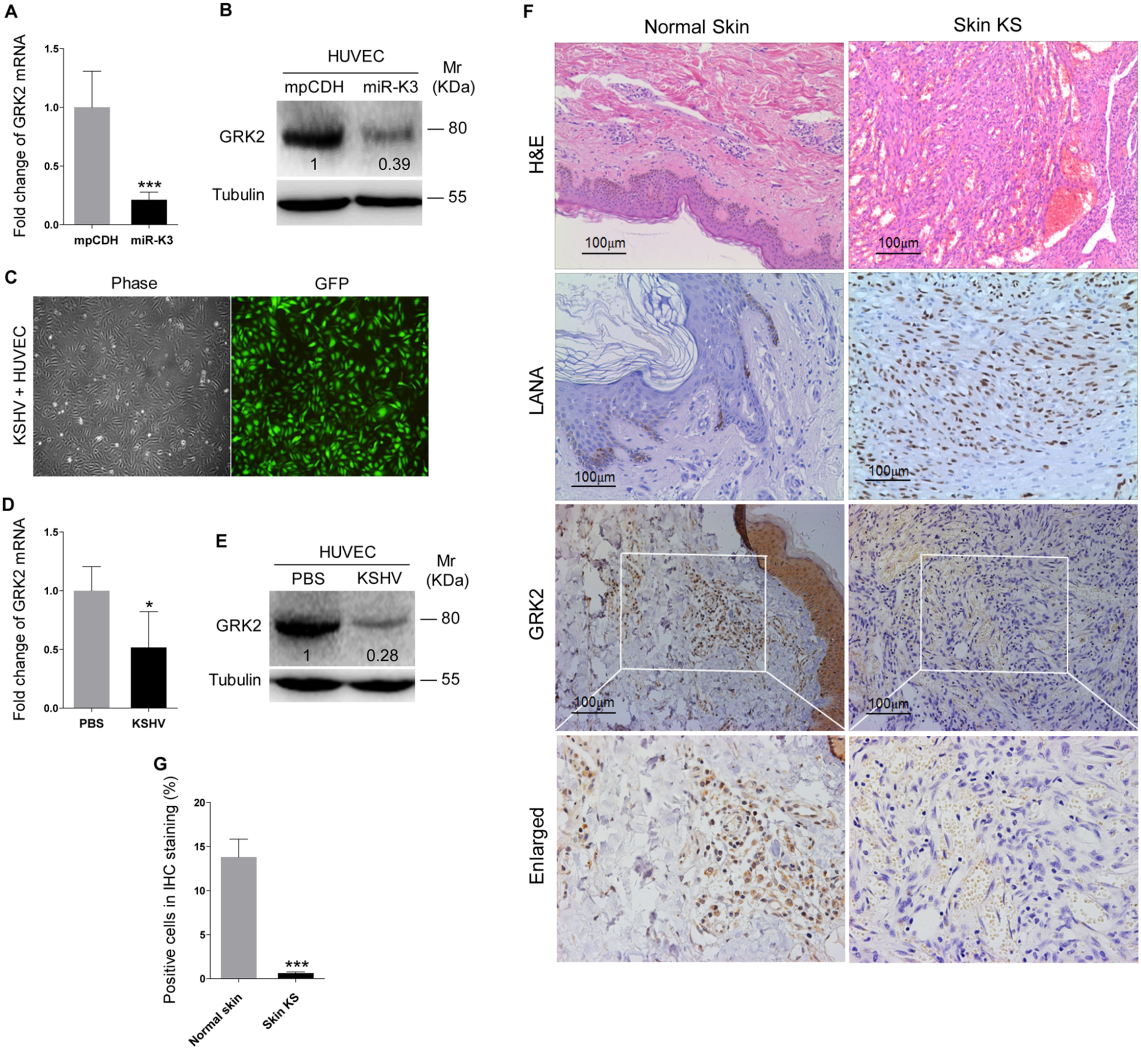
GRK2 expression is reduced in miR-K3-expressing HUVEC and KS lesion samples. **(A)**. The mRNA level of GRK2 in HUVEC transduced with lentivirus empty vector (**mpCDH**) and lentivirus-miR-K3 (**miR-K3**) were examined by qPCR. **(B)**. The expression of GRK2 proteins in HUVEC transduced with lentivirus empty vector (**mpCDH**) and lentivirus-miR-K3 (**miR-K3**) were detected by Western blotting analysis. Results shown were from a representative experiment of three independent experiments with similar results. The values of density of protein bands after normalization to housekeeping were shown; same for all of the following Western blotting figures. **(C)**. HUVEC were infected with KSHV, and image captured under light microscope (**Phase**) and fluorescent microscope (**GFP**) (Original magnification, ×100). **(D)**. qPCR analysis for GRK2 mRNA in KSHV-infected HUVEC as described in (**C**) (**KSHV**) or in HUVEC treated with PBS as the negative control (**PBS**). **(E)**. Western blotting analysis of the expression of GRK2 protein in KSHV-infected HUVEC as described in (**C**) (**KSHV**) or in HUVEC treated with PBS as the negative control (**PBS**). **(F)**. Hematoxylin and eosin (H&E) staining of KS lesion (**right**) and normal (**left**) tissue sections to show histologic features (left panel; original magnification, ×100) and immunohistochemical staining (IHC) of KSHV LANA and GRK2 (middle and left panels; original magnification, ×200). **(G)**. Quantification of results in (**F**). *** *P* < 0.001 for Student’s *t*-test.

Since miRNAs usually exert their functions by binding to their target genes to induce the degradation of the transcripts or inhibit the translation of proteins, bioinformatics analysis with several programs including TargetScan, RNAhybrid, Findtar, and Pita, was performed to predict the putative miR-K3 targets. Based on complementarity with the seed sequences of miR-K3, a putative binding site was predicted in the 3’UTR of G protein-coupled receptor kinase 2 (GRK2), which was also predicted as a potential target of miR-K3 in the previous studies [45,46]. qPCR and Western blotting analyses indeed showed that both mRNA and protein of GRK2 were markedly down-regulated in miR-K3-expressing HUVEC compared to that of the vector control cells (Fig 2A and 2B). To determine whether KSHV infection can decrease GRK2 expression, we examined HUVEC infected with KSHV (Fig 2C). As expected, both mRNA and protein levels of GRK2 were also dramatically reduced in KSHV-infected HUVEC (Fig 2D and 2E). Consistent with these observations, there were less GRK2-postive cells in the KS lesions compared to the normal skin tissues as shown by immunohistochemistry staining (Fig 2F and 2G).

**Fig 3:**
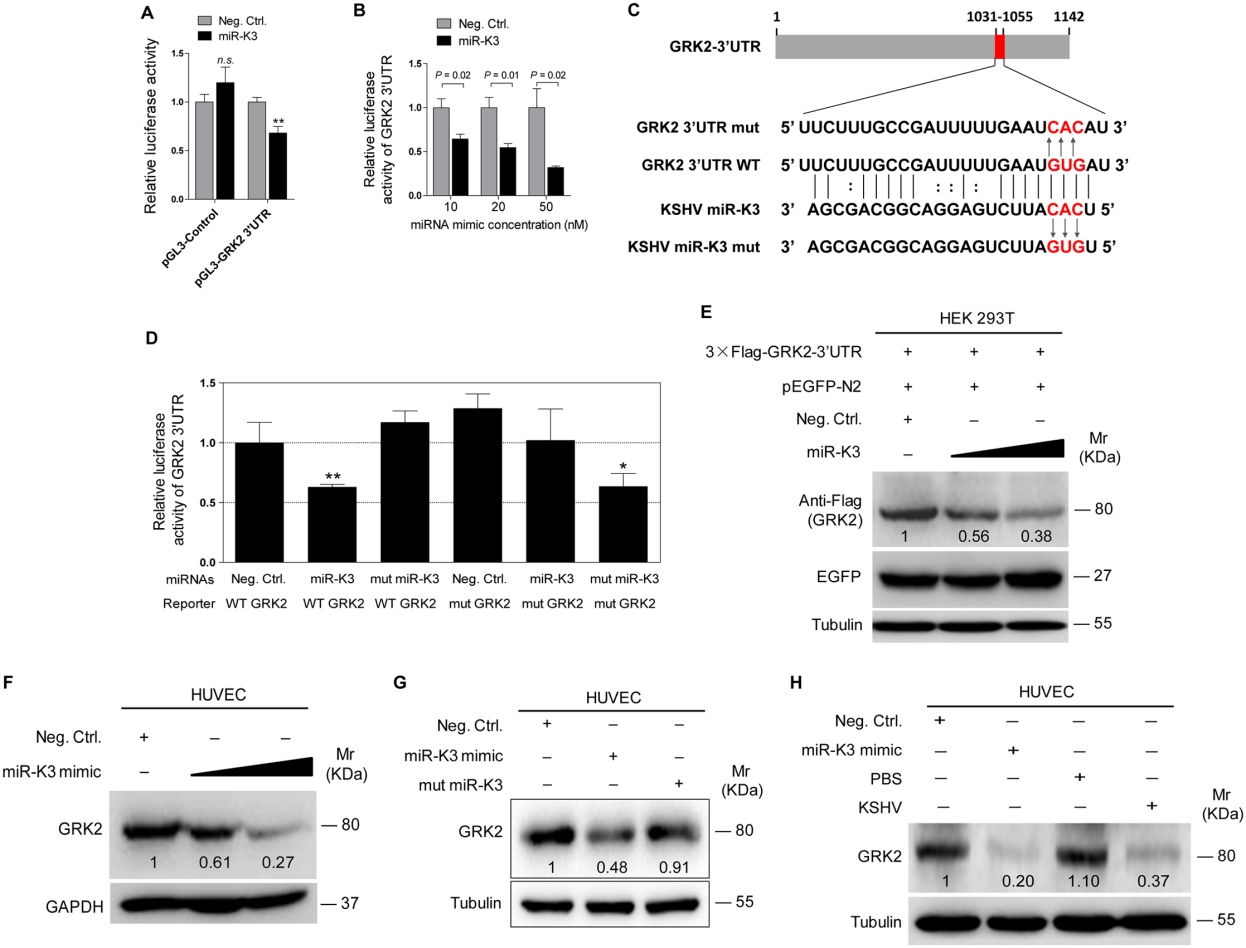
GRK2 is directly targeted by miR-K3. **(A)**. Luciferase activity was detected in HEK 293T cells co-transfected by a mimic of miR-K3 (**miR-K3**) or a negative control nucleotide of miRNA (**Neg. Ctrl.**) together with pGL3-Control or pGL3-GRK2 3’UTR luciferase reporter (**pGL3-GRK2 3’UTR**). ** *P* < 0.01 for Student’s *t*-test. *n*.*s*., not significant. **(B)**. Luciferase assay of 293T cells co-transfected by pGL3-GRK2 3′UTR together with increasing amounts (10, 20, and 50 nM) of miR-K3. **(C)**. Schematic illustration of the putative seed sequences of miR-K3 complementary with GRK2 3’UTR and mutagenesis of binding sites in the 3’UTR of GRK2. **(D)**. The luciferase activity was assayed in 293T cells co-transfected by GRK2 wild type 3’UTR (**WT GRK2**) or the mutant GRK2 3’UTR construct (**mut GRK2**) together with **miR-K3** or mutant miR-K3 mimic (**mut miR-K3**). * *P* < 0.05 and ** *P* < 0.01 for Student’s *t*-test. **(E)**. miR-K3 inhibited the expression of exogenous GRK2 protein by targeting its native 3’UTR. Western blotting was performed in HEK 293T cells co-transfected by pcDNA3.1–3×Flag-GRK2-3’UTR together with pEGFP and increasing amounts (10 and 20 nM) mimic of miR-K3. **(F)**. miR-K3 inhibited the expression of endogenous GRK2 protein in HUVEC transfected with increasing amounts (10 and 20 nM) mimic of miR-K3. **(G)**. Mutant miR-K3 failed to target endogenous GRK2. Western blotting was performed in HUVEC transfected by Neg. Ctrl., miR-K3 mimic (20 nM) or mut miR-K3 lacking the seed sequences. **(H)**. Transfection of miR-K3 mimic (20 nM) has the same inhibition level on GRK2 expression as that of KSHV infection.

To validate GRK2 as a direct target of miR-K3, the full length 3’UTR of GRK2 was amplified and inserted into the downstream of luciferase sequence in the pGL3-Control plasmid (named as pGL3-GRK2 3’UTR). The luciferase reporter assays indicated that miR-K3 significantly inhibited the GRK2 3’UTR reporter activity in a dose-dependent manner but not the pGL3-Control construct (Fig 3A and 3B). We identified a miR-K3 seed sequence in the GRK2 3’UTR (Fig 3C). Mutation of this seed sequence abolished the inhibitory effect of miR-K3 on GRK2 3’UTR (Fig 3D). To directly evaluate the effect of miR-K3 on GRK2 protein expression, miR-K3 mimic was transiently co-transfected with GRK2 expression plasmid, pcDNA3.1–3×Flag-GRK2-3’UTR containing the native 3’UTR, into HEK 293T cells. Western blotting showed that miR-K3 strongly attenuated the expression of GRK2 in a dose-dependent fashion (Fig 3E). Importantly, miR-K3 mimic also markedly suppressed the expression of endogenous GRK2 in HUVEC in a dose-dependent manner (Fig 3F) while the mutant mimic of miR-K3 lacking the seed sequences did not (Fig 3G). The expression level of GRK2 in cells transfected with miR-K3 mimic was similar to that of KSHV infection (Fig 3H). Together these data suggest that GRK2 is a direct target of miR-K3.

### MiR-K3 Induces Endothelial Cell Migration and Invasion by Targeting GRK2

**Fig 4:**
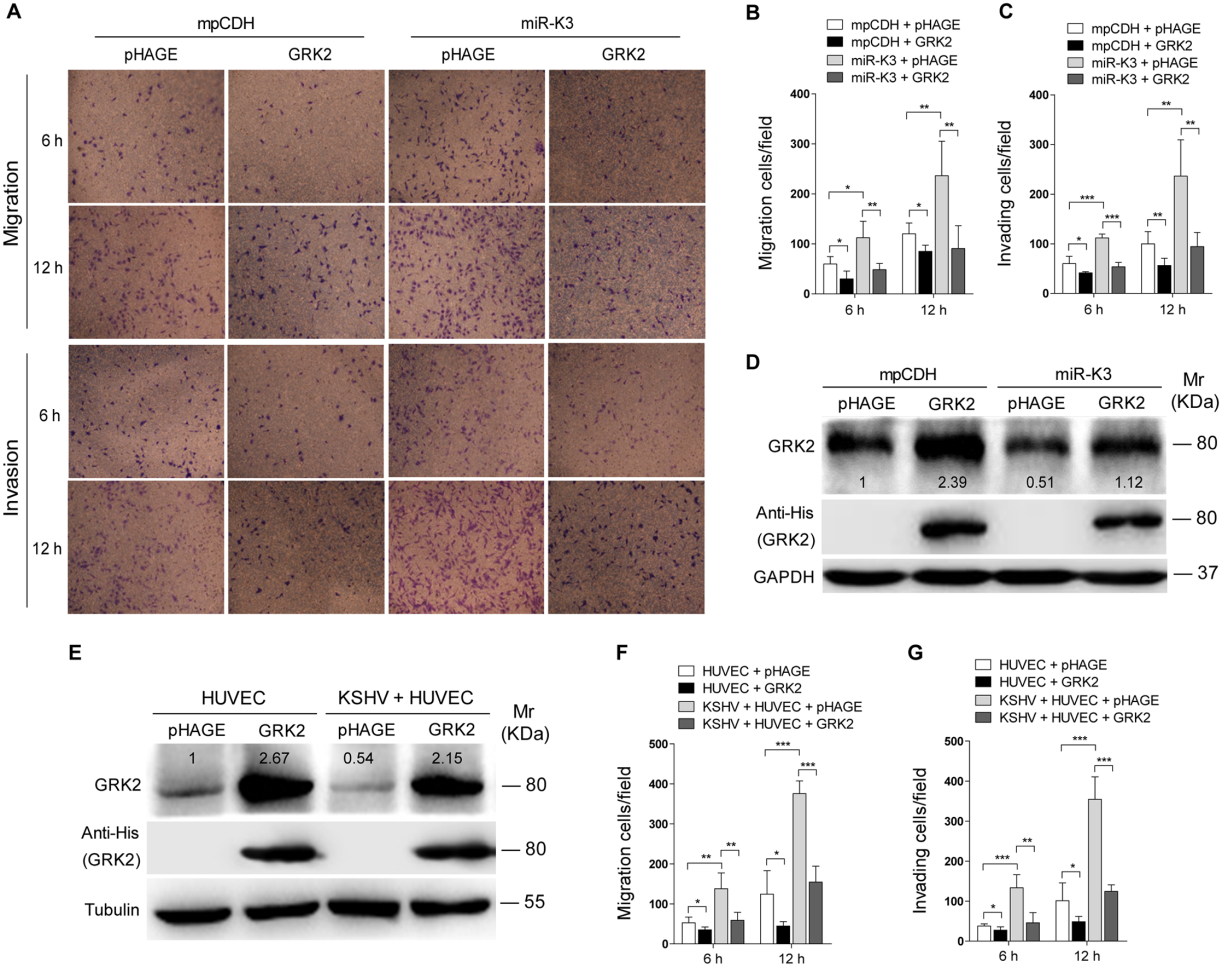
Ectopic expression of GRK2 inhibits miR-K3-induced endothelial cell migration and invasion. **(A)**. Transwell migration (top) and Matrigel invasion (bottom) assays for HUVEC transduced with lentivirus-mediated empty vector (**mpCDH**) or miR-K3 (**miR-K3**), which were subsequently co-transduced with lentivirus-mediated empty vector (**pHAGE**) and lentivirus-GRK2 (**GRK2**), respectively. The representative images were captured at 6 and 12 h post seeding (original magnification, ×100). **(B)**. The quantification results of Transwell migration assay in (**A**). * *P* < 0.05, ** *P* < 0.01 and *** *P* < 0.001 for Student’s *t*-test. **(C)**. The quantification results of Matrigel invasion assay in (**A**). ** *P* < 0.01 and *** *P* < 0.001 for Student’s *t*-test. **(D)**. Western blotting was performed in HUVEC treated as in (**A**) with the indicated antibodies. The antibody against His-tag was used to detect the exogenous expression of GRK2. **(E)**. Western blotting was performed in normal HUVEC transduced with lentivirus-GRK2 (**GRK2**) and its control (**pHAGE**), or KSHV-infected HUVEC transduced with lentivirus-GRK2 (**GRK2**) and its control (**pHAGE**) with the indicated antibodies. The antibody against His-tag was used to examine the exogenous expression of GRK2. **(F)**. Transwell migration assay for HUVEC treated as in (**E**) at 6 and 12 h post seeding. * *P* < 0.05, ** *P* < 0.01 and *** *P* < 0.001 for Student’s *t*-test. **(G)**. Matrigel invasion assay for HUVEC treated as in (**E**) at 6 and 12 h post seeding. * *P* < 0.05, ** *P* < 0.01 and *** *P* < 0.001 for Student’s *t*-test.

Decrease of GRK2 has been shown to augment the migratory response of polymorphonuclear leukocytes (PMNs) [47]. To characterize the role of GRK2 in miR-K3-induced migration and invasion of HUVEC, miR-K3-expressing HUVEC were transduced with lentivirus-GRK2. Overexpression of GRK2 significantly abolished miR-K3-induced cell migration and invasion at 6 h and 12 h post-seeding (Fig 4A, 4B and 4C), Western-blotting confirmed the suppression of endogenous GRK2 by miR-K3 (Lane 3 *vs* lane 1 in Fig 4D). Transduction with lentivirus-GRK2 increased the expression level of GRK2 but was reduced by miR-K3 (Lane 2 *vs* lane 4 in Fig 4D). As expected, KSHV infection also downregulated the expression of endogenous GRK2 (Lane 3 *vs* lane 1 in Fig 4E). Again, transduction with lentivirus-GRK2 increased the expression level of GRK2 but was reduced by KSHV infection (Lane 2 *vs* lane 4 in Fig 4E). Consistent with these results, while KSHV infection enhanced cell migration and invasion, overexpression of GRK2 inhibited cell migration and invasion of both HUVEC and KSHV-infected HUVEC (Fig 4F and 4G).

**Fig 5:**
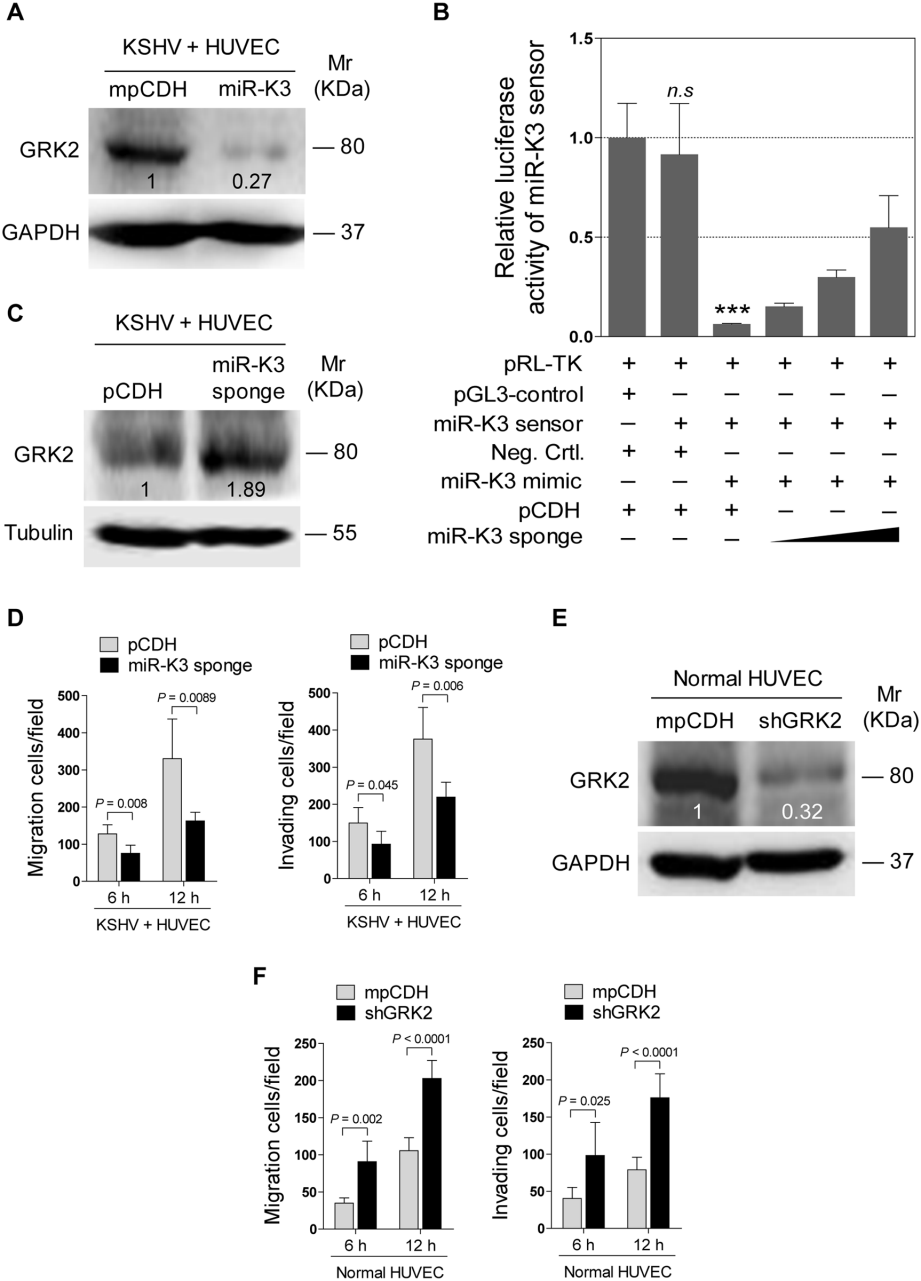
KSHV infection promotes endothelial cell migration and invasion through miR-K3 by targeting GRK2. **(A)**. Western blotting was performed in KSHV-infected HUVEC (**KSHV + HUVEC**) transduced with lentivirus empty vector (**mpCDH**) or lentivirus-miR-K3 (**miR-K3**) with the indicated antibodies. **(B)**. MiR-K3 sponge was functional. HEK 293T cells were co-transfected with miR-K3 sensor reporter and miR-K3 mimic, and subsequently transduced with increasing MOI of lentivirus-mediated miR-K3 sponge (**miR-K3 sponge**) or its control (**pCDH**). The cells were collected at 48 h post-transduction for luciferase assays. *** *P* < 0.001 for Student’s *t*-test. *n*.*s*., not significant. **(C)**. Western blotting was performed in KSHV-infected HUVEC (**KSHV + HUVEC**) transduced with miR-K3 sponge (**miR-K3 sponge**) or its control (**pCDH**) with the indicated antibodies. **(D)**. Transwell migration (**Left panel**) and Matrigel invasion (**Right panel**) assays for cells treated as in (**C**) at 6 and 12 h post seeding. **(E)**. Western blotting was performed in normal HUVEC transduced with lentivirus-mediated a mixture of short hairpin RNAs targeting GRK2 (**shGRK2**) or the control (**mpCDH**) with the indicated antibodies. **(F)**. Transwell migration (**Left panel**) and Matrigel invasion (**Right panel**) assays for cells treated as in (**E**) at 6 and 12 h post seeding.

In addition, overexpression of miR-K3 in KSHV-infected HUVEC reduced the expression of GRK2 (Fig 5A) and further enhanced cell migration and invasion (S2 Fig). To further confirm the role of miR-K3 targeting in KSHV-induced cell migration and invasion, we generated a miR-K3 sponge. In the luciferase reporter assay, transduction of the sponge abolished the inhibitory effect of miR-K3 mimic on its sensor reporter in a dose-dependent manner in HEK 293T cells, indicating that the miR-K3 sponge was functional (Fig 5B). Transduction of the miR-K3 sponge into KSHV-infected HUVEC increased the expression level of GRK2 (Fig 5C) and inhibited cell migration and invasion (Fig 5D). As expected, knock-down of GRK2 by lentivirus-mediated a mixture of short hairpair RNAs in normal HUVEC alone was sufficient to increase cell migration and invasion (Fig 5E and 5F, S3 Fig). Collectively, these results indicated that KSHV-induced cell migration and invasion was mediated by miR-K3 targeting of GRK2.

### GRK2 Mediates MiR-K3-Induced Cell Migration and Invasion through the CXCR2/AKT Pathway

**Fig 6:**
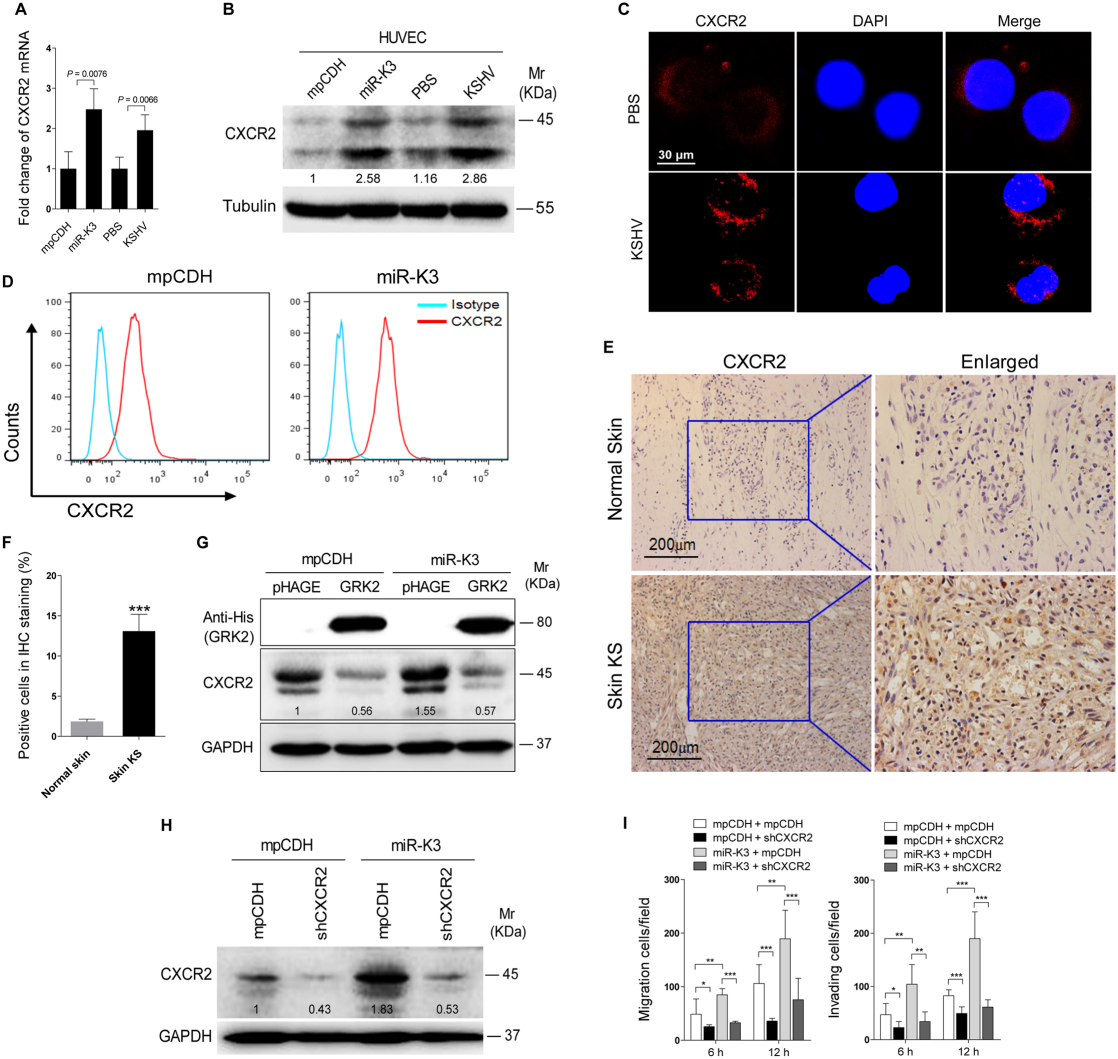
Activation of CXCR2, which was negatively regulated by GRK2, contributes to miR-K3-induced endothelial cell migration and invasion. **(A)**. The mRNA level of CXCR2 in HUVEC transduced with lentivirus empty vector (**mpCDH**) and lentivirus-miR-K3 (**miR-K3**) or HUVEC treated with PBS (**PBS**) and infected with KSHV (**KSHV**) were examined by qPCR. **(B)**. The expressions of CXCR2 protein in HUVEC treated as in (**A**). **(C)**. Confocal microscopy of HUVEC treated as in (A), then stained for red fluorescence protein (refers to CXCR2; red). 4’, 6’-diamidino-2-phenylindole (DAPI) (blue) stains nuclei. **(D)**. Representative flow cytometry histograms for CXCR2 expression on the surface of HUVEC treated as in (A). Cells were stained with anti-CXCR2 MAb and fluorescein isothiocyanate-labeled IgG was used as an isotype control antibody. **(E)**. Immunohistochemical (IHC) staining of CXCR2 in normal skin (**Normal Skin**; top) and KS lesions (**Skin KS**; bottom). **(F)**. Quantification of the results in (**E**). *** *P* < 0.001 for Student’s *t*-test. **(G)**. Western blotting was performed using HUVEC transduced with lentivirus-mediated miR-K3 (**miR-K3**) or empty vector (**mpCDH**), and co-transduced with GRK2 (**GRK2**) or its control (**pHAGE**), respectively, with the indicated antibodies. The antibody against His-tag was used to detect the exogenous GRK2. **(H)**. Western blotting was performed in HUVEC transduced with miR-K3 (**miR-K3**) or empty vector (**mpCDH**), which were further transduced with a mixture of short hairpin RNAs targeting CXCR2 (**shCXCR2**) or its control (**mpCDH**), respectively. **(I)**. Transwell migration (left) and Matrigel invasion (right) assays were performed in HUVEC treated as in (**E**). * *P* < 0.05, ** *P* < 0.01 and *** *P* < 0.001 for Student’s *t*-test.

It has been reported that GRK2 was negatively correlated with the expression of the chemokine receptor CXCR2 in neutrophils, and increased expression of GRK2 down-regulated CXCR2, leading to impairment of neutrophil migration into an infectious focus *in vivo* [48,49]. Given these findings, we reasoned that CXCR2 may also be involved in GRK2 mediation of miR-K3-induced cell migration and invasion. Indeed, both mRNA and protein levels of CXCR2 were elevated in miR-K3-expressing and KSHV-infected HUVEC compared to the respective control cells (Fig 6A and 6B). In agreement with its membrane localization, we observed a higher level of CXCR2 on the membrane of KSHV-infected HUVEC than mock infected control cells (Fig 6C). Similar results were also observed on the surface of HUVEC transected with a miR-K3 mimic (S4 Fig). As expected, flow cytometry analysis showed a higher level of CXCR2 surface expression on miR-K3-transduced HUVEC than on the cells transduced with the control vector (Fig 6D). Importantly, we observed a higher level of CXCR2 expression in KS lesions than the normal skin tissues by immunohistochemistry staining (Fig 6E and 6F). To determine whether the increased expression of CXCR2 in the miR-K3-expressing cells was due to the downregulation of GRK2, we overexpressed GRK2 in the miR-K3-expressing HUVEC. As shown in Fig 6G, overexpression of GRK2 dramatically down-regulated CXCR2 expression in both normal and miR-K3-expressing HUVEC. To determine the role of CXCR2 in miR-K3-mediated cell migration and invasion, we performed knock-down of CXCR2 with lentivirus-mediated a mixture of short hairpair RNAs (shCXCR2) (Fig 6H and S5 Fig). Knock-down of CXCR2 significantly inhibited miR-K3-induced cell migration and invasion (Fig 6I). These data indicated that CXCR2 mediated miR-K3 induced cell migration and invasion as a result of miR-K3 targeting of GRK2.

**Fig 7:**
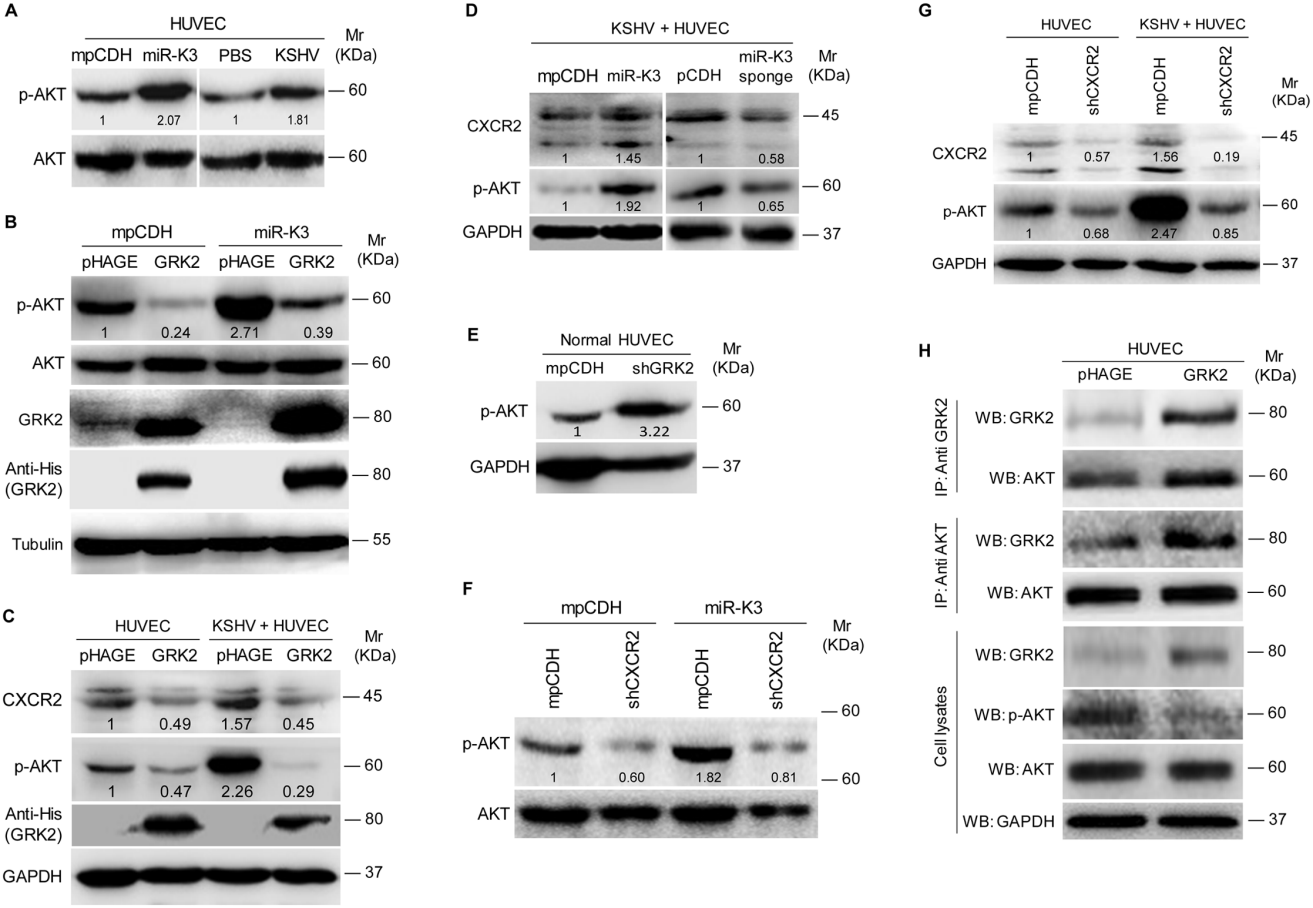
MiR-K3 enhances the activation of AKT in HUVEC by targeting GRK2. **(A)**. Western blotting analysis of phosphorylated and total AKT in HUVEC transduced with **mpCDH** or **miR-K3**, and HUVEC treated with PBS (**PBS**) or infected with KSHV (**KSHV**), respectively. **(B)**. Western blotting analysis of phosphorylated AKT in HUVEC transduced with **mpCDH** or **miR-K3**, which were further transduced with lentivirus-GRK2 (**GRK2**) or its control (**pHAGE**). **(C)**. Western blotting analysis for CXCR2 and phosphorylation levels of AKT in normal HUVEC or KSHV-infected HUVEC transduced with lentivirus-GRK2 (**GRK2**) or its control (**pHAGE**). **(D)**. Western blotting analysis of CXCR2 and the phosphorylation levels of AKT in KSHV-infected HUVEC transduced with lentivirus-mediated miR-K3 (**miR-K3**) and its control (**mpCDH**) or lentivirus-mediated miR-K3 sponge (**miR-K3 sponge**) and its control (**pCDH**), respectively. **(E)**. Western blotting analysis of phosphorylation levels of AKT in normal HUVEC transduced with lentivirus-mediated a mixture of short hairpin RNAs targeting GRK2 (**shGRK2**) or the control (**mpCDH**). **(F)**. Western blotting analysis of phosphorylation levels of AKT in HUVEC transduced with lentivirus-mediated miR-K3 (**miR-K3**) or empty vector (**mpCDH**), and further with lentivirus-mediated a mixture of short hairpin RNAs targeting CXCR2 (**shCXCR2**). **(G)**. Western blotting analysis of phosphorylated AKT in normal HUVEC or KSHV-infected HUVEC transduced with lentivirus-mediated a mixture of short hairpin RNAs targeting CXCR2 (**shCXCR2**) or its control (**mpCDH**). **(H)**. GRK2 physiologically hijacked AKT in GRK2-expressing HUVEC. HUVEC were transduced with lentivirus-GRK2 (**GRK2**) or its control (**pHAGE**) and subjected to co-immunoprecipitation with the antibody against GRK2 (**IP: Anti GRK2**) or AKT (**IP: Anti AKT**) followed by Western blotting using indicated antibodies.

Since CXCR2 activated AKT signaling to promote the migration and invasion of lymphocytes and cancer cells [50,51], we asked whether AKT signaling was also involved in miR-K3 and KSHV induction of cell migration and invasion. Consistent with the previous reports [52], KSHV infection of HUVEC induced the phosphorylation of AKT (Fig 7A). Expression of miR-K3 also induced the phosphorylation of AKT in HUVEC (Fig 7A). Overexpression of GRK2 in miR-K3-expressing HUVEC dramatically inhibited AKT activation (Fig 7B). Similar results were also observed in KSHV-infected HUVEC, where ectopic expression of GRK2 led to the inhibition of AKT activation and a reduction of CXCR2 level (Fig 7C). In addition, overexpression of miR-K3 further enhanced AKT activation and increased the expression level of CXCR2 in KSHV-infected HUVEC while miR-K3 sponge effectively reduced the levels of phosphorylated AKT and CXCR2 expression (Fig 7D). As expected, knockdown of GRK2 with shRNAs was sufficient to increase the phosphorylated AKT level in normal HUVEC (Fig 7E). Because the expression level of CXCR2 was positively correlated with the level of AKT activation (Fig 7C and 7D), we examined the role of CXCR2 in AKT activation. Knock-down of CXCR2 decreased the level of phosphorylated AKT in either miR-K3-expressing or KSHV-infected HUVEC (Fig 7F and 7G). These observations implied that miR-K3/GRK2/CXCR2/AKT axis mediates miR-K3- and KSHV-induced cell migration and invasion.

It has been reported that GRK2 interacts with AKT and inhibits its activation to regulate endothelial cell nitric oxide synthase function in portal hypertension [53]. We examined the physiological interaction between GRK2 and AKT in the current system. Co-immunoprecipitation assay indeed showed that GRK2 interacted with AKT (Fig 7H). Overexpression of GRK2 increased the amount of GRK2-immunoprecipiated AKT and reduced the level of activated AKT (Fig 7H). These data indicated that, besides increasing the level of CXCR2 to activate AKT, low expression of GRK2 as a result of miR-K3 targeting released AKT, resulting in higher level of AKT activation.

**Fig 8:**
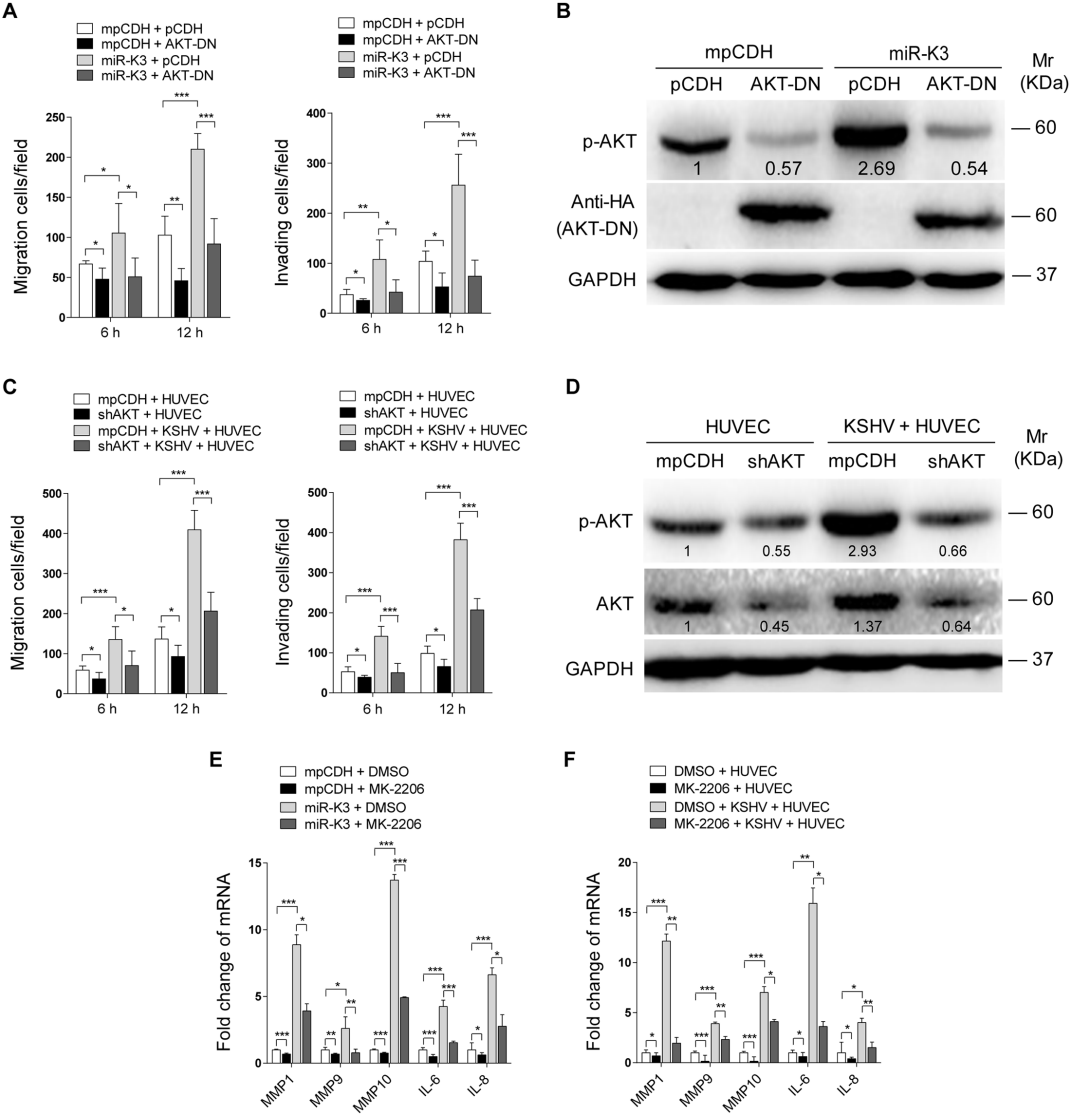
Activation of AKT is necessary to miR-K3-induced endothelial cell migration and invasion. **(A)**. Transwell migration (**Left panel**) and Matrigel invasion (**Right panel**) assays for HUVEC which were transduced with lentivirus-mediated empty vector (**mpCDH**) or miR-K3 (**miR-K3**) expression and further with lentivirus-AKT-DN (**AKT-DN**) or its control (**pCDH**). * *P* < 0.05, ** *P* < 0.01 and *** *P* < 0.001 for Student’s *t*-test. **(B)**. Western blotting analysis of phosphorylated AKT in HUVEC treated as in (**A**). The antibody against HA-tag was used to detect the transduction of AKT-DN. **(C)**. Transwell migration (**Left panel**) and Matrigel invasion (**Right panel**) assays for KSHV-infected HUVEC transduced with lentivirus-mediated a mixture of short hairpin RNAs targeting AKT (**shAKT**) or its control (**pCDH**). * *P* < 0.05 and *** *P* < 0.001 for Student’s *t*-test. **(D)**. Western blotting analysis of phosphorylated AKT levels in HUVEC treated as in (**C**). **(E)**. The mRNA expression of MMP1, 9, 10 and IL-6, 8 in HUVEC, which were transduced with lentivirus-mediated empty vector (**mpCDH**) or miR-K3 (**miR-K3**) expression and further treated with the AKT inhibitor, MK-2206 (**MK-2206**) or its control (**DMSO**), were determined by qPCR. * *P* < 0.05, ** *P* < 0.01 and *** *P* < 0.001 for Student’s *t*-test. **(F)**. The mRNA expression of MMP1, 9, 10 and IL-6, 8 in KSHV-infected HUVEC treated with the AKT inhibitor, MK-2206 (**MK-2206**) or its control (**DMSO**) were determined by qPCR. * *P* < 0.05, ** *P* < 0.01 and *** *P* < 0.001 for Student’s *t*-test.

Our results showed that both GRK2/CXCR2/AKT signaling and GRK2/AKT interaction could lead to higher level of AKT activation. To determine if miR-K3-induced AKT activation mediated the enhanced cell migration and invasion, we transduced miR-K3-expressing HUVEC with a dominant negative mutant of AKT (AKT-DN). Expression of AKT-DN inhibited cell migration and invasion in miR-K3-expressing HUVEC (Fig 8A). Western blotting confirmed the decreased level of phosphorylated AKT (Fig 8B). Similar inhibition of cell migration and invasion was also observed in KSHV-infected HUVEC following knock down of AKT (Fig 8C and 8D, S6 Fig). To further confirm these observations, MK-2206, an AKT inhibitor, was used to treat miR-K3-transduced or KSHV-infected HUVEC. Consistently, treatment of MK-2206 not only inhibited cell migration and invasion (S7A and S7C Fig), but also decreased the level of phosphorylated AKT (S7B and S7D Fig). Importantly, inhibition of AKT with MK-2206 also blocked the induction of MMP1, 9 and 10, and IL-6, IL-8 by miR-K3 transduction or KSHV infection in HUVEC (Fig 8E and 8F).

Taken together, these data suggest that KSHV and miR-K3 promote endothelial cell migration and invasion by targeting GRK2 through activation of the CXCR2/AKT pathway.

### Deletion of miR-K3 from the KSHV Genome Attenuates KSHV-Induced Cell Migration and Invasion

**Fig 9:**
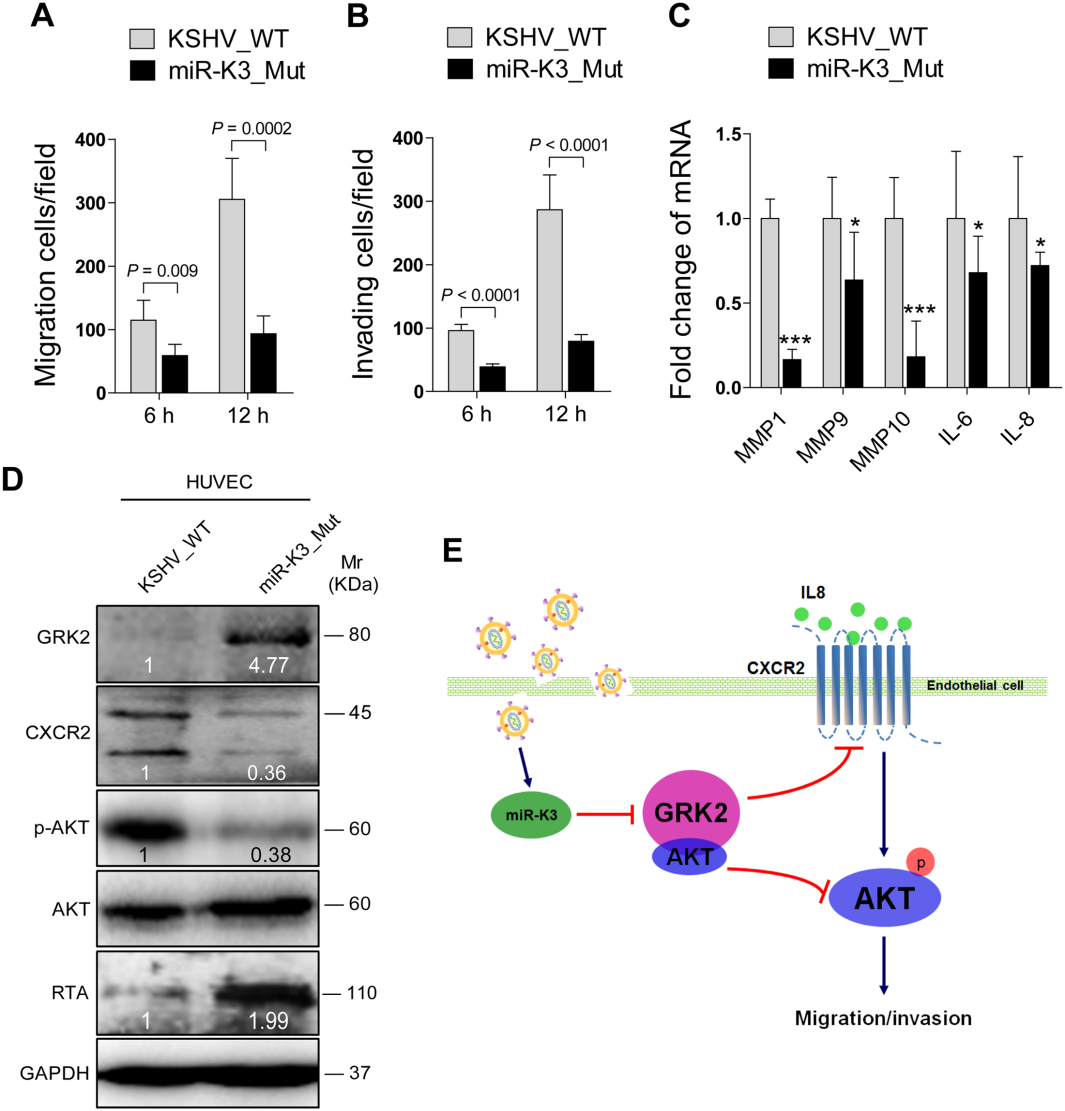
Deletion of miR-K3 from the KSHV genome attenuates KSHV induction of endothelial cell migration and invasion. **(A)**. Transwell migration assay for HUVEC infected with BAC16 KSHV wide type virus (**KSHV_WT**) or BAC16 KSHV miR-K3 deletion mutant virus (**miR-K3_Mut**). * *P* < 0.05, ** *P* < 0.01 and *** *P* < 0.001 for Student’s *t*-test. **(B)**. Matrigel invasion assay for HUVEC treated as in (**A**). * *P* < 0.05, ** *P* < 0.01 and *** *P* < 0.001 for Student’s *t*-test. **(C)**. The mRNA expression of MMP1, 9, 10 and IL-6, 8 in HUVEC treated as in (**A**) were determined by RT-qPCR. **(D)**. Western blotting analysis of expression of GRK2, CXCR2, phosphorylated AKT, and KSHV RTA in HUVEC treated as in (**A**) with the indicated antibodies. **(E)**. Schematic representation of the mechanism by which miR-K3 facilitates endothelial cell migration and invasion.

Since inhibition of miR-K3 function with a specific sponge in KSHV infected HUVEC decreased cell migration and invasion (Fig 5D), we wished to further confirm these results by genetic knock out of miR-K3 from the KSHV genome. We next infected HUVEC with wild type recombinant KSHV BAC16 and a mutant with miR-K3 deleted (BAC16 miR-K3_mut virus). We ensured that the expression of miR-K3 was abrogated in miR-K3_mut virus and the deletion of miR-K3 from the KSHV genome didn’t affect the expression of other miRNAs by qPCR analysis (S8 Fig). As shown in Fig 9A and 9B, the levels of cell migration and invasion in HUVEC infected by the mutant virus were significantly lower than those infected by the wild type virus. Consistent with these observations, cells infected by the mutant virus had decreased levels of MMP1, 9, and 10 and IL-6 and IL-8 mRNAs transcripts than those infected by the wild type virus (Fig 9C). Importantly, mutant cells had a higher level of GRK2, and lower levels of CXCR2 and activated AKT than those of wild type cells (Fig 9D), while RTA was dramatically elevated in mutant cells (Fig 9D), which was consistent with previous reports [20,22]. To further confirm the role of AKT in miR-K3 regulation of mRNAs transcripts of MMPs and inflammatory cytokines, we transfected AKT cDNA into HUVEC infected by the mutant virus. As expected, transfection of AKT cDNA in miR-K3_mut-infected HUVEC increased the levels of MMP1, 9, and 10 and IL-6 and IL-8 transcripts compared to cells transfected with control vector (S9 Fig). These data collectively suggest that during promotion of cell migration and invasion, miR-K3 up-regulates the levels of transcripts of MMPs and inflammatory cytokines through the activation of AKT signaling.

## Discussion

Cell migration and invasion play a central role in tumor progression and metastasis. Acquisition of a migratory or invasive phenotype represents one of the hallmarks of KSHV infected endothelial cells, which might also be the underlying basis of tumor dissemination and angiogenesis in KS tumors. Previous studies have shown that KSHV infection promotes invasion of human primary endothelial cells *in vitro* [6]. KSHV infection also increased cell invasion by inducing multiple matrix metalloproteinases (MMPs), including MMP-1, MMP-2, and MMP-9, and disrupting the cell-cell and cell-matrix interactions [6]. KSHV-encoded latent nuclear antigen (LANA), Kaposin B, and K15 have been shown to regulate cells migration and invasion by modulating several cellular cancer-related miRNAs including the miR-221/miR-222 cluster and miR-31, which target ETS2 and ETS1, and the migration inhibitor FAT4, respectively [43,44]. Other studies also showed that expression of KSHV-encoded G protein-coupled receptor (vGPCR) and interleukin-6 (vIL-6) conferred cells with pro-migratory and pro-invasive phenotypes [54,55]. However, there has been no study to examine the roles of KSHV-encoded miRNAs in cell migration and invasion.

miR-K3 has been shown to regulate viral life cycle [22,56]. Interestingly, it is also expressed at a high level in KS lesions [57], suggesting its potential role in KS pathogenesis. In this study, we have shown that expression of miR-K3 alone or infection by KSHV promotes cell migration and invasion of HUVEC. Importantly, either suppression of miR-K3 function with a sponge or deletion of miR-K3 from the KSHV genome inhibited KSHV-induced cell migration and invasions in endothelial cells. As tumorigenesis is involved with a highly complex series of steps, including (I) sustained proliferative signaling, (II) evasion of growth suppression, (III) activated invasion and metastasis, (IV) enabled replicative immortality, (V) induced angiogenesis and (VI) resistance to cell death [58], our results have provided strong evidences that miR-K3 may play an important role in the invasion and metastasis of KSHV-related malignancies.

As a serine/threonine kinase in the regulation of multiple GPCRs, GRK2 specifically recognizes and phosphorylates agonist-activated GPCRs to trigger intracellular signaling cascades [59,60]. The two previous studies by using PAR-CLIP and ago HITS-CLIP, respectively, showed that GRK2 with the official gene symbol *ADRBK1* was predicted as a potential target of miR-K3 [45,46]. Consistently, in this study, using the bioinformatics analysis including TargetScan, RNAhybrid, Findtar, and Pita, we also predicted GRK2 as a potential target of miR-K3. More importantly, we subsequently revealed that GRK2 was directly targeted by miR-K3 and mediated miR-K3-induced cell migration and invasion. GRK2 has been reported to positively regulate epithelial cell migration [61]; however, down-modulation of GRK2 also reduces chemokine receptor desensitization and augments the migratory response of polymorphonuclear leukocytes (PMNs) [47]. Additionally, decreased GRK2 in endothelial cells promoted chemoattraction to VEGF [62]. In line with the established role of GRK2 in cell migration of endothelial cells and immune cells, we have also shown that GRK2 negatively regulates miR-K3-induced migration and invasion of endothelial cells. Therefore, the effect of the altered GRK2 expression on cell migration might be dependent on the cell types and possibly other physiological contexts [47,63–66].

In fact, down-modulation of GRK2 levels increases chemotactic responses to different agonists that are reminiscent of induction of immune cell migration, whereas enhanced expression of GRK2 attenuates chemotaxis, which is consistent with its canonical negative regulatory role in GPCR signaling [67]. Our results indicate that miR-K3 induction of cell migration through targeting of GRK2 is linked to the chemokine receptor CXCR2, a seven transmembrane type GPCR. The CXCR2, alternatively known as IL-8RB, is critical for the recruitment of neutrophils from the circulation to the site of infection [68]. In addition to being expressed in neutrophils, CXCR2 is also found in mast cells, monocytes and macrophages. In non-immune cells, CXCR2 is found in endothelial cells where it mediates angiogenesis [69]. In epithelial cells and multiple tumor cells, CXCR2 is induced by activated oncogenes [69]. Previous studies showed that GRK2 negatively regulated CXCR2 during neutrophil migration to sites of inflammation [48,49]. Our results showed that GRK2 was downregulated while CXCR2 was upregulated in miR-K3-expressing HUVEC, KSHV-infected HUVEC, and KS lesion tissues. Consistently, deletion of K3 from the KSHV genome not only restored the expression of GRK2 but also reduced the expression of CXCR2, resulting in a lower activated AKT level and attenuated cell migration and invasion of KSHV infected endothelial cells. These data imply that by expressing miR-K3, KSHV targets GRK2 to activate CXCR2 signaling, leading to enhanced cell migration and invasion, which might contribute to KS progression. Since KSHV-encoded more than two dozens of miRNAs, our results did not exclude the possibility that, by regulating other targets, other viral miRNAs may also be involved in KSHV-induced cell migration and invasion.

Hyper-activation of the AKT signaling is characteristic of almost all human malignancies, including KS and PEL [70,71]. Indeed, KSHV infection activates the AKT signaling in B cells and endothelial cells [52]. Several KSHV-encoding genes including ORF-K1, vGPCR, vIL-6 and ORF45 have been shown to induce AKT signaling [52,72–74]. In this study, we found that overexpression of KSHV miR-K3 alone led to elevated AKT signaling in HUVEC, while suppression of the activity of miR-K3 with the sponge construct or deletion of miR-K3 from the KSHV genome significantly decreased the phosphorylation level of AKT in KSHV-infected endothelial cells, indicating the contribution of miR-K3 to aberrant AKT signaling during KSHV infection. Further, up-regulation of CXCR2 is correlated with activated AKT signaling during the migration of lymphocytes and tumor progression of cancer cells [50,51]. In agreement with these reports, we showed that knock-down of CXCR2 dramatically inhibited AKT activation, leading to attenuation of miR-K3 induction of cell migration and invasion. These results support our hypothesis that activation of CXCR2/AKT signaling promoted KSHV-induced cell migration and invasion via miR-K3 downregulation of GRK2. In fact, besides inhibition of the canonical CXCR2/AKT pathway, GRK2 has been reported to directly interact with AKT through its C-terminus (aa 492–689) to inhibit AKT activation [53]. Our co-immunoprecipitation results showed that GRK2 interacted with AKT and inhibited AKT activation in miR-K3-expressing and KSHV-infected endothelial cells. miR-K3 downregulation of GRK2 released AKT and led to its activation resulting in the enhanced cell migration and invasion. Therefore, in addition to regulation of KSHV induction of angiogenesis, deregulation of cellular energetics, evasion of growth suppression and lymphatic reprogramming [72,75,76], our results also reveal a novel role of AKT signaling in the invasion and metastasis of KSHV-related malignancies.

In summary, we have found that miR-K3 promotes the migration and invasion of KSHV-infected endothelial cells by targeting the GRK2/CXCR2/AKT and GRK2/AKT signaling pathways (Fig 9E). These findings further illustrate the complex regulatory networks of KSHV miRNAs and their multiple targets. Considering the role of miR-K3 in inhibiting KSHV lytic replication and its high expression level during viral latency and in KS tumors, it would be interesting to further delineate the role of miR-K3/GRK2/AKT axis in KSHV-induced angiogenesis and tumorigenesis.

## Materials and Methods

### Ethics Statement

The clinical section of the research was reviewed and ethically approved by the Institutional Ethics Committee of the First Affiliated Hospital of Nanjing Medical University (Nanjing, China).

### Cell Culture

Primary human umbilical vein endothelial cells (HUVEC) were isolated from the interior of the umbilical vein of human umbilical cords by digestion with collagenase (Sigma, St. Louis, MO, USA) as described [77]. HUVEC were cultured in complete EBM-2 culture media (LONZA, Allendale, NJ, USA) and used between passage 3 and 6. HEK 293T were cultured as previously described [78]. All cells were cultured at 37°C in a humidified, 5% CO_2_ atmosphere.

### Plasmids

The wild type GRK2 3’UTR reporter construct (GRK2 3’UTR WT) and its mutant (GRK2 3'UTR mut) were made by cloning the full length of the GRK2 3’UTR sequence and the same sequence but with a mutation in the miR-K3 targeting seed sequence into the downstream of the luciferase sequence in the pGL3-Control plasmid (Promega, Shanghai, China), respectively. The lentiviral transferring plasmid, pHAGE-CMV-MCS-IzsGreen (named as pHAGE), was used in this study as previously described [79,80]. Human GRK2 gene expressed with a His tag at the C-terminus was amplified using the cDNA from HUVEC as PCR templates and inserted into pHAGE to generate recombinant pHAGE-GRK2 for production and transduction of lentivirus. The expressing plasmid of GRK2 with a 3x flag tags and the full 3’UTR sequences of GRK2 was cloned into pcDNA3.1 vector and designated as pcDNA3.1–3×Flag-GRK2-3’UTR for transfection in HEK 293T cells. The plasmid pEGFP-N2 from CLONTECH Laboratories (Palo Alto, CA, USA) was used to calibrate the efficiency of transfection. The pGL3-miR-K3 sensor reporter named as pGL3-miR-K3 Sensor was constructed by stitching three tandem miR-K3 seed binding sequences and inserted into the downstream of luciferase sequence in the pGL3-Control plasmid. The sequence is as following: (forward) 5’-TCG CTG CCG TCC TCA GAA TGT GAA AGC TTT CGC TGC CGT CCT CAG AAT GTG AAA GCT TTC GCT GCC GTC CTC AGA ATG TGA-3’ and (reverse) 5’-TCA CAT TCT GAG GAC GGC AGC GAA AGC TTT CAC ATT CTG AGG ACG GCA GCG AAA GCT TTC ACA TTC TGA GGA CGG CAG CGA-3’. The miR-30 based microRNA (miR-K3) expressing plasmid was constructed by two steps: First, the miR-30 precursor stem-loops plus RFP coding sequences were amplified from the pTRIPZ plasmid (Open Biosystems, AL, USA) and inserted into another lentiviral plasmid pCDH-CMV-MCS-EF1-copGFP (System Bioscience, CA, USA) to create a new lentiviral plasmid, which has both GFP and RFP cassettes and was designed as modified pCDH (mpCDH). Second, the precursor stem-loops of miR-K3 was amplified using primers (forward) 5’-CAG AAG GCT CGA GAA GGT ATA TTG CTG TTG ACA GTG AGC G-3’ and (reverse) 5’-CTA AAG TAG CCC CTT GAA TTC CGA GGC AGT AGG CA-3’) and cloned into mpCDH. Meanwhile, the mpCDH plasmid was also used as the short hairpin RNA (shRNA) expressing lentiviral vector. The short hairpin RNA (shRNA) complementary sequences to GRK2, CXCR2, and AKT were listed in Table 1. MiR-K3 sponge, which antagonizes the miR-K3, was constructed by stitching six tandem sponge sequences with the annealing primers (forward) 5’-AAT TTT CGC TGC CGT CAG TGA ATG TGA CGA TTC GCT GCC GTC AGT GAA TGT GAA CCG GTT CGC TGC CGT CAG TGA ATG TGA GAA TTC CGG CGG -3’ and (reverse) 5’- GAT CCC GCC GGA ATT CTC ACA TTC ACT GAC GGC AGC GAA CCG GTT CAC ATT CAC TGA CGG CAG CGA ATC GTC ACA TTC ACT GAC GGC AGC GAA -3’), and then inserted into pCDH-CMV-MCS-EF1-copGFP (pCDH) as described elsewhere [81]. The dominant negative construct of AKT (designated as AKT-DN) [79] carried a HA flag was also cloned into pCDH. The pCMV3-HA-AKT construct containing AKT cDNA and its corresponding control pCMV3-C-HA were purchased from Sino Biological Inc. (Beijing, China). In this study, the control of pHAGE-GRK2 was named as pHAGE and the controls of miR-K3 and all the shRNA were a modified pCDH (mpCDH for short); while the controls of miR-K3 sponge and AKT-DN were designed as pCDH.

**Table 1:** The sequences of the shRNAs.

Gene	shRNA No.	Sequence of shRNA (5’to 3’)
GRK2	sh1	ATGCAGACAATGAATGGGC
	sh2	AATCTCCGGTTGACATCCC
	sh3	TTTGTGTCCTCCTCATCGA
CXCR2	sh1	TTTCCAGGGATTCTGGTTC
	sh2	ATCTTGAGGAGTCCATGGC
	sh3	ATTGTTGCCCATGTCCTCA
AKT	sh1	ACACCTCCATCTCTTCAGC
	sh2	TTGGCCACGATGACTTCCTTC
	sh3	TCCTGGTTGTAGAAGGGCAGG

### BAC16 and miR-K3 Mutant BAC16ΔmiR-K3

Wild type recombinant KSHV BAC16 and a KSHV mutant with miR-K3 deleted, BAC16 ΔmiR-K3, were previously described [22,82].

### Transfection and Luciferase Reporter Assay

Transfections of HUVEC were performed with the Effectence transfection reagent (Qiagen, Valencia, CA, USA), while other transfections were performed with Lipofectamine 2000 (Invitrogen, Carlsbad, CA, USA) following the manufacturer’s instructions.

For luciferase assay, HEK 293T cells (1×10^5^) were transfected with miRNA mimic, luciferase reporter DNA and Renilla vector pRL-TK (Promega, Madison, WI), and then harvested at 48 h post-transfection. Relative luciferase activity was assayed using the Promega dual-luciferase reporter assay system.

### Antibodies, Western Blotting and Reagents

Anti-KSHV LANA ORF73 rat monoclonal antibody (MAb) was purchased from Advanced Biotechnologies Inc. (Columbia, MD, USA) [78]. Anti-phospho-AKT (Ser473) rabbit MAb, anti-AKT rabbit polyclonal antibody (PAb), anti-Flag M2 rabbit MAb, anti-His rabbit MAb and anti-HA rabbit PAb were obtained from Cell Signaling Technologies (Beverly, MA, USA). Anti-GRK2 mouse MAb, anti-CXCR2 rabbit PAb, anti-GAPDH mouse MAb, anti-α-Tubulin mouse MAb, and horseradish peroxidase (HRP)-conjugated goat anti-mouse or anti-rabbit IgG were all purchased from Santa Cruz Biotechnology (Santa Cruz, CA, USA). Anti-GFP mouse MAb was from Beyotime Institute of Biotechnology (Nantong, Jiangsu, China). Western blotting analysis was performed as described previously [83]. MK-2206, an AKT inhibitor, was purchased from Selleck Chemicals (Shanghai, China).

### Production of BAC16 Virus Stock

Production of KSHV BAC16 virus was performed according to the previous study [82]. Briefly, stable iSLK-BAC16 or BAC16ΔmiR-K3 cells were treated with both doxycycline (1 μg/ml) and sodium butyrate (1 mM). Two days later, the old medium was removed and replaced with maintaining medium. Another two or three days later, cell supernatant was collected and cleared of cells and debris by centrifugation (3,000 rpm for 10 min at room temperature) and filtration (0.45 μm). Virus particles were pelleted by ultracentrifugation of the cell supernatant through 20% sucrose cushion (24,000 rpm for 3 h at 4°C) using an SW32 Ti rotor. The supernatant was then discarded and the virus pellet was re-suspended in a desired volume.

### Production and Transduction of Lentivirus

To obtain the recombinant lentivirus, the virus-packaging cells HEK 293T were seeded for 24 h later and then co-transfected with lentiviral plasmids, packaging vector psPAX2 and envelope vector pMD2.G as previously described [81]. The virus containing supernatants were collected 48 h after transfection.

### Transwell Migration and Matrigel Invasion Assay

Cell migration was measured using Millicell hanging cell culture inserts with 8-μm-pore polyethylene terephthalate membrane filter (Merck Millipore, Darmstadt, Germany). Cell invasiveness was examined similarly to Transwell migration assay, except that the Millicell hanging cell culture inserts were coated with 40 μl Matrigel mixed with 20 μl basic medium at first [6]. Typically, for migration or invasion assay, cells were seeded at 1×10^5^/well in the upper chambers with 200 μl serum-free basic medium. Then, 500 μl complete endothelial cell growth medium (EGM-2 with growth factors) was placed in the lower wells serving as a source of chemoattractants. The cells were incubated for 6 h and 12 h at 37°C. Cells migrated to the lower surface of the filter were fixed with 70% methanol and stained with 0.5% crystal violet solution. The number of migrated cells was determined by counting stained cells and the average cell number per field for each well was calculated. The counting was blinded by three individuals, including one who was blinded to the results. For each experiment, three to five replicate wells were used and the representative images were taken from five randomly selected fields of each well.

### Wound Healing Assay

mpCDH or miR-K3-transduced HUVEC were plated in six-well plates and grown to confluence. The next day medium was removed, and wounds were introduced by scraping the confluent cell cultures with a 200 μl pipette tip. Floating cells were carefully removed before the addition of complete medium. The wound-healing process then was monitored with an inverted light microscopy (Olympus, Japan) immediately after wounding (0 h), and again at 12 h and 36 h. The distance between the edges of the wound were measured at ten different areas from the wound edge to edge. The measurements were then converted into a percentage using the following formula: % of wound remaining = (measurement at time 12 hours/measurement at time 0 hours) × 100; then the percentage of wound closure was calculated as follows: 100%–% of wound remaining.

### RNA Isolation and Real-Time Quantitative Reverse Transcription-PCR (qPCR)

Total RNA was isolated from cells by Trizol reagent (Invitrogen, Carlsbad, CA, USA) and subjected to the Promega Reverse-Transcription Kit (Promega, Madison, WI) to obtain cDNA. The sequences of specific primers of RT-qPCR for several genes were listed in Table 2. Quantitative PCR (qPCR) was performed using SYBR *Premix Ex Taq* Kit (TaKaRa Biotechnology Co. Ltd., Dalian, China) according to the manufacturer’s instructions.

**Table 2:** The sequences of specific primers for qPCR.

Target	Application	Primer
GRK2	RT-qPCR	F: 5′-ATGCATGGCTACATGTCCAA -3′
		R: 5′-ATCTCCTCCATGGTCAGCAG -3′
CXCR2	RT-qPCR	F: 5′- ACAGAGAGTTGGGAGCCACT -3′
		R: 5′- GGGCATGCCAGAGCTATAAT -3′
MMP1	RT-qPCR	F: 5′- AATGTGCTACACGGATACCC -3′
		R: 5′- CTTTGTGGCCAATTCCAGGA -3′
MMP9	RT-qPCR	F: 5′- CGGAGTGGCAGGGGGAAGATGCTG -3′
		R: 5′- GCAGGATGTCATAGGTCACGTAGC -3′
MMP10	RT-qPCR	F: 5′- GGACCTGGGCTTTATGGAGATAT -3′
		R: 5′- CCCAGGGAGTGGCCAAGT-3′
IL-6	RT-qPCR	F: 5′- GAAAGGAGACATGTAACAAGAGT -3′
		R: 5′- GATTTTCACCAGGCAGTCT -3′
IL-8	RT-qPCR	F: 5′- ACCACCGGAAGGAACCATTC -3′
		R: 5′- TTCACACAGAGCTGCAGAAATCA -3′
GAPDH	RT-qPCR	F: 5′-GAAGGTGAAGGTCGGAGTC -3′
		R: 5′-GAAGATGGTGATGGGATTTCC -3′

### Immunohistochemistry (IHC)

The KS clinical tissue specimens and the normal skin tissue specimens were provided by the First Affiliated Hospital of Nanjing Medical University for hematoxylin-eosin staining (H&E) and IHC. All the samples were formalin-fixed, parafin-embedded, and immunostained with the indicated antibodies as previously described [81]. The results were processed and analyzed using Image-Pro Plus 6.0 image analysis system (Media Cybernetics, Silver Spring, MD). Five random fields were chosen under the microscope and further measured for area and intensity of the expression of target protein, with the expression level of target protein calculated based on average absorbance (gray).

### Immunofluorescence Assay (IFA)

Cells were plated in chamber slides and incubated at 37°C overnight for attachment. The cells in the chamber slides were washed 3 times in PBS and fixed in cold acetone for 15 min at room temperature and then washed twice with PBS for 5 min. After washing, the cells were blocked with 0.05% BSA in PBS at 37°C for 20 min. The primary antibody CXCR2 rabbit PAb (1:100 dilution) was applied in the slides at 4°C overnight. And then the slides were washed 3 times with PBS and incubated with the second antibody Alexa Fluor 555 donkey anti-rabbit (1:200 dilution) (Beyotime Institute of Biotechnology, Nantong, China) at 37°C for 45 min. Finally, the slides were washed 3 times with PBS and counterstained with DAPI and observed with a Zeiss Axiovert 200 M epifluorescence microscope (Carl Zeiss, Inc.).

### Flow Cytometry

To detect the expression of CXCR2 on the cell surface, 1×10^6^ cells were collected and suspended in cold PBS, then incubated with APC-conjugated mouse anti-human CXCR2 MAb and the isotype control APC mouse IgG1 on ice for 30 min (BD Biosciences, USA), respectively. Cells were then washed with cold PBS and analyzed with the FACScan (BD Biosciences, USA).

### Co-immunoprecipitation

For total cell lysates, immunoprecipitation was performed using a protocol modified from previous studies [53]. Briefly, HUVEC were transduced with lentivirus-GRK2 or its control. After 72 h post-transduction, HUVEC were collected, rinsed twice with cold PBS, and lysed in Lysis/Wash buffer (150 mM NaCl, 1 mM EDTA, 5% glycerol, 1% NP-40, 25 mM Tris-HCl, pH7.4) supplemented with protease inhibitors, phosphatase inhibitors and phenylmethylsulfonyl fluoride (PMSF). Lysates were cleared by centrifugation at 11,000 g at 4°C for 10 min. Supernatants were incubated with 5 μg of the specified antibody overnight at 4°C followed by 50 μl of protein A-beads for 4 h at 4°C with gentle rotation. The beads were then pelleted at 5,000 g for 2 min and washed 3 times in 1 ml ice-cold Lysis/Wash buffer containing 1 mM PMSF and 50 g/ml aprotinin. Antibody-protein conjugates were eluted by boiling (5 min) and samples were then subjected to SDS-PAGE and immunoblotting as described above.

## Supporting Information

S1 Table A list of accession numbers/ID numbers for genes mentioned in the text. (DOCX)

S2 Table A list of accession numbers/ID numbers for miRNAS mentioned in the text. (DOCX)

S1 Fig Ectopic expression of miR-K3 promotes endothelial cell migration. Wound healing assays were performed in HUVEC transduced with lentivirus empty vector (**mpCDH**) or lentivirus-miR-K3 (**miR-K3**). The representative images were captured at 0, 12, and 36 h post seeding (original magnification, ×100).
(TIF)

S2 Fig Overexpression of miR-K3 in KSHV infected HUVEC further promotes cell migration and invasion. Transwell migration (**Left panel**) and Matrigel invasion (**Right panel**) assays for KSHV-infected HUVEC (**KSHV + HUVEC**) transduced with lentivirus empty vector (**mpCDH**) or lentivirus-miR-K3 (**miR-K3**) at 6 and 12 h post seeding.
(TIF)

S3 Fig Screening and identification of lentivirus-mediated short hairpin RNA targeting GRK2. Western blotting was performed in HUVEC transduced with lentivirus-mediated No.1 (**sh1GRK2**), No. 2 (**sh2GRK2**), No. 3 (**sh3GRK2**), and a mixture of No. 1, 2, and 3 together (**shGRK2**) of short hairpin RNAs targeting GRK2 or the control (**mpCDH**) with the indicated antibodies.
(TIF)

S4 Fig The expression of CXCR2 protein in miR-K3 expressing-HUVEC. Confocal microscopy of HUVEC transfected by a mimic of miR-K3 (**miR-K3**) or a negative control nucleotide of miRNA (**Neg. Ctrl.**), then stained for red fluorescence protein (refers to CXCR2; red). 4’, 6’-diamidino-2-phenylindole (DAPI) (blue) stains nuclei.
(TIF)

S5 Fig Screening and identification of lentivirus-mediated short hairpin RNA targeting CXCR2. Western blotting was performed in HUVEC transduced with lentivirus-mediated No.1 (**sh1CXCR2**), No. 2 (**sh2CXCR2**), No. 3 (**sh3CXCR2**), and a mixture of No. 1, 2, and 3 together (**shCXCR2**) of short hairpin RNAs targeting CXCR2 or the control (**mpCDH**) with the indicated antibodies.
(TIF)

S6 Fig Screening and identification of lentivirus-mediated short hairpin RNA targeting AKT. Western blotting was performed in HUVEC transduced with lentivirus-mediated No.1 (**sh1AKT**), No. 2 (**sh2AKT**), No. 3 (**sh3AKT**), and a mixture of No. 1, 2, and 3 together (**shAKT**) of short hairpin RNAs targeting AKT or the control (**mpCDH**) with the indicated antibodies.
(TIF)

S7 Fig Activation of AKT is necessary for miR-K3-induced endothelial cell migration and invasion. **(A)**. Transwell migration (**Left panel**) and Matrigel invasion (**Right panel**) assays for HUVEC which were transduced with lentivirus-mediated empty vector (**mpCDH**) or miR-K3 (**miR-K3**) expression and further treated with the AKT inhibitor, MK-2206 (**MK-2206**) or its control (**DMSO**). * *P* < 0.05, ** *P* < 0.01 and *** *P* < 0.001 for Student’s *t*-test. **(B)**. Western blotting analysis of phosphorylated AKT in HUVEC treated as in (**A**). **(C)**. Transwell migration (**Left panel**) and Matrigel invasion (**Right panel**) assays for KSHV-infected HUVEC treated with the AKT inhibitor, MK-2206 (**MK-2206**) or its control (**DMSO**). * *P* < 0.05, ** *P* < 0.01 and *** *P* < 0.001 for Student’s *t*-test. **(D)**. Western blotting analysis of phosphorylated AKT levels in HUVEC treated as in (**C**).
(TIF)

S8 Fig Deletion of miR-K3 from the KSHV genome doesn’t affect the expression of other miRNAs. Total RNA was extracted from HUVEC infected with BAC16 KSHV wide type virus (**KSHV_WT**) or BAC16 KSHV miR-K3 deletion mutant virus (**miR-K3_Mut**), and levels of KSHV miRNAs miR-K3, -K4-3p, -K6-3p, and -K6-5p were measured by using qPCR. undet, undetermined. *n*.*s*., not significant.
(TIF)

S9 Fig MiR-K3 up-regulates the levels of transcripts of MMPs and inflammatory cytokines through the activation of AKT signaling. Total RNA was extracted from the BAC16 KSHV wide type virus (**KSHV_WT**)- or BAC16 KSHV miR-K3 deletion mutant virus (**miR-K3_Mut**)-infected HUVEC, which were further transfected with pCMV3-HA-AKT construct (**AKT cDNA**) or its corresponding control pCMV3-C-HA (**pCMV**). The mRNA expression of MMP1, 9, 10 and IL-6, 8 were determined by qPCR.
(TIF)
