# Supplementary material for: A KSHV microRNA Directly Targets G Protein-Coupled Receptor Kinase 2 to Promote the Migration and Invasion of Endothelial Cells by Inducing CXCR2 and Activating AKT Signaling
Source: PLoS Pathog. 2015 Sep 24;11(9):e1005171. doi: 10.1371/journal.ppat.1005171 (PMC4581863; doi:10.1371/journal.ppat.1005171)
Supplement: S1 Table — (DOCX) [file ppat.1005171.s001.docx]

**S1 Table**. A list of accession numbers/ID numbers for genes mentioned in the text.

| Gene name | GENE ID |
| --- | --- |
| GRK2 | 156 |
| CXCR2 | 3579 |
| MMP1 | 4312 |
| MMP2 | 4313 |
| MMP9 | 4318 |
| MMP10 | 4319 |
| IL6 | 3569 |
| IL8 | 3576 |
| AKT | 207 |
| FAT4 | 79633 |
| VEGF | 7422 |
| NFIB | 4781 |
| MYB | 4602 |
| C/EBPα | 140815 |
| Ets-1 | 2113 |
| Ets-2 | 2114 |
| KSHV LANA | 4961527 |
| KSHV Kaposin B | 4961446 |
| KSHV K15 | 4961473 |
| KSHV vGPCR | 4961465 |
| KSHV vIL6 | 4961449 |
| KSHV K1 | 4961511 |
| KSHV ORF45 | 4961474 |
| KSHV RTA | 4961526 |
