# Supplementary material for: A KSHV microRNA Directly Targets G Protein-Coupled Receptor Kinase 2 to Promote the Migration and Invasion of Endothelial Cells by Inducing CXCR2 and Activating AKT Signaling
Source: PLoS Pathog. 2015 Sep 24;11(9):e1005171. doi: 10.1371/journal.ppat.1005171 (PMC4581863; doi:10.1371/journal.ppat.1005171)
Supplement: S2 Table — (DOCX) [file ppat.1005171.s002.docx]

**S2 Table**. A list of accession numbers/ID numbers for miRNAS mentioned in the text.

| microRNA | Accession number |
| --- | --- |
| KSHV-miR-K3 | MIMAT0002193 |
| KSHV-miR-K5 | MIMAT0002190 |
| KSHV-miR-K7-5p | MIMAT0015217 |
| KSHV-miR-K9-5p | MIMAT0002184 |
| KSHV-miR-K11 | MIMAT0002181 |
| hsa-miR-221 | MIMAT0000278 |
| hsa-miR-222 | MIMAT0000279 |
| hsa-miR-31 | MIMAT0000089 |
