# Supplementary figures and images for: A KSHV microRNA Directly Targets G Protein-Coupled Receptor Kinase 2 to Promote the Migration and Invasion of Endothelial Cells by Inducing CXCR2 and Activating AKT Signaling
Source: PLoS Pathog. 2015 Sep 24;11(9):e1005171. doi: 10.1371/journal.ppat.1005171 (PMC4581863; doi:10.1371/journal.ppat.1005171)

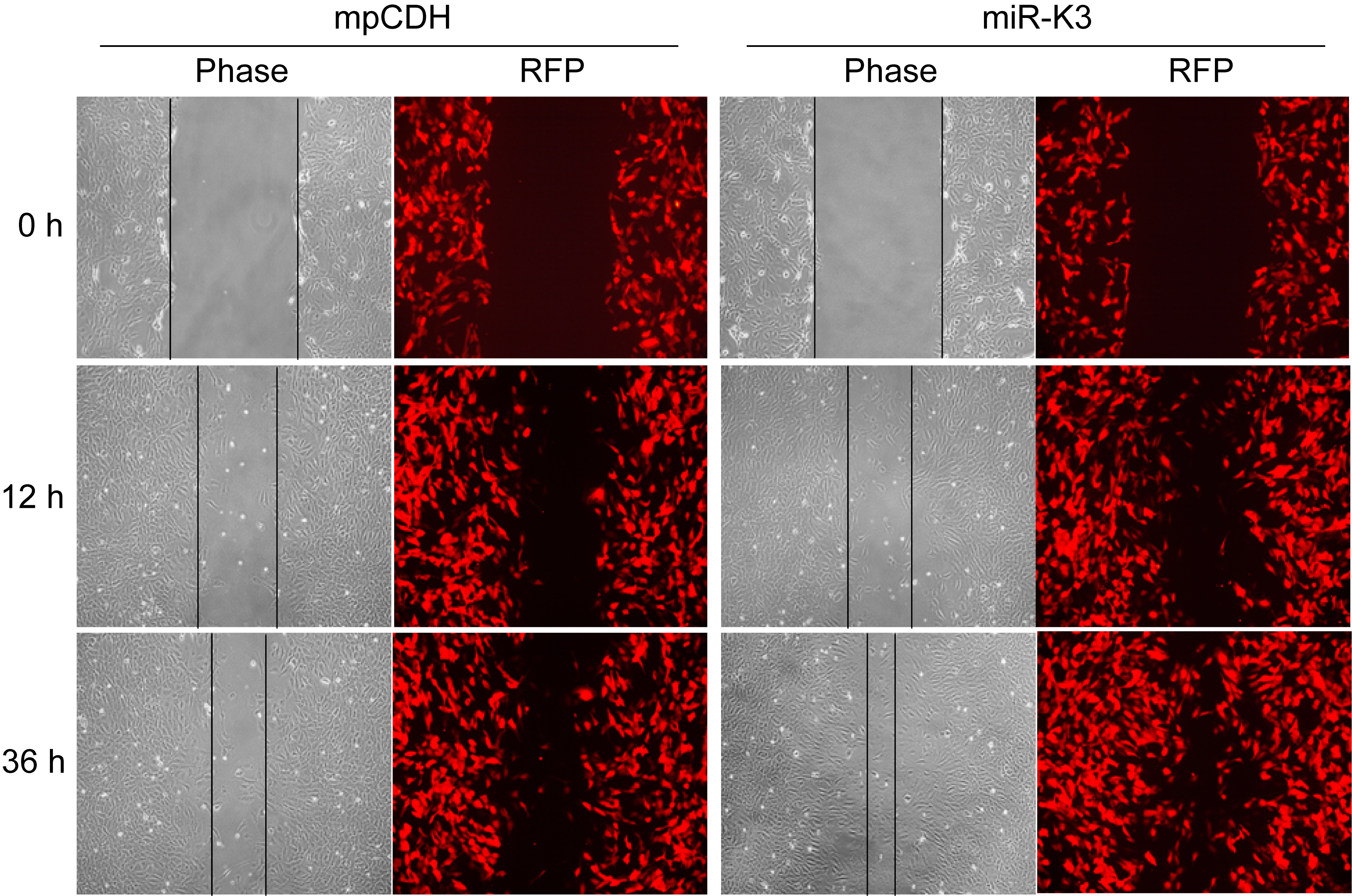

Supplement: S1 Fig — Wound healing assays were performed in HUVEC transduced with lentivirus empty vector (mpCDH) or lentivirus-miR-K3 (miR-K3). The representative images were captured at 0, 12, and 36 h post seeding (original magnification, ×100). (TIF) [file ppat.1005171.s003.tif]

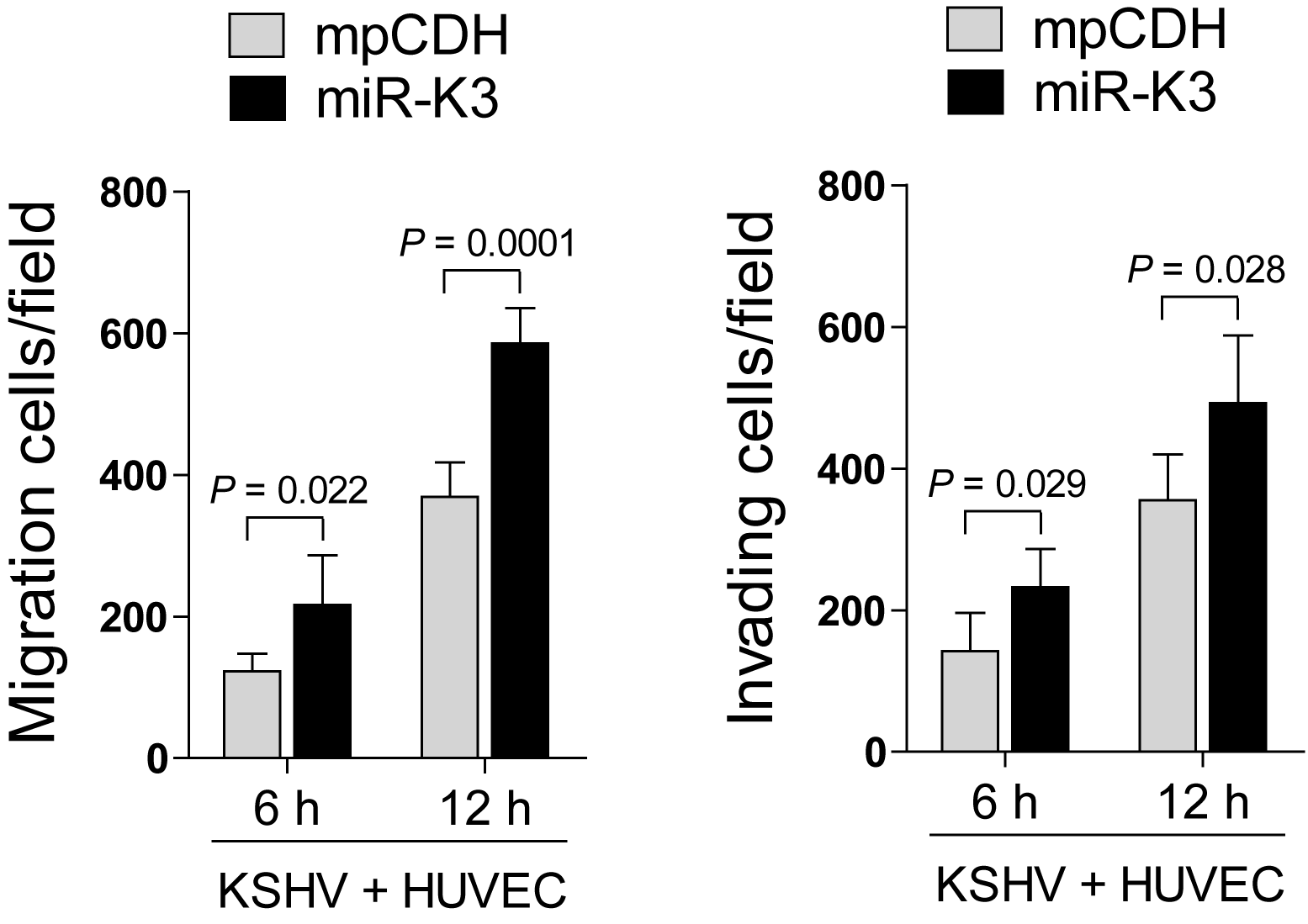

Supplement: S2 Fig — Transwell migration (Left panel) and Matrigel invasion (Right panel) assays for KSHV-infected HUVEC (KSHV + HUVEC) transduced with lentivirus empty vector (mpCDH) or lentivirus-miR-K3 (miR-K3) at 6 and 12 h post seeding. (TIF) [file ppat.1005171.s004.tif]

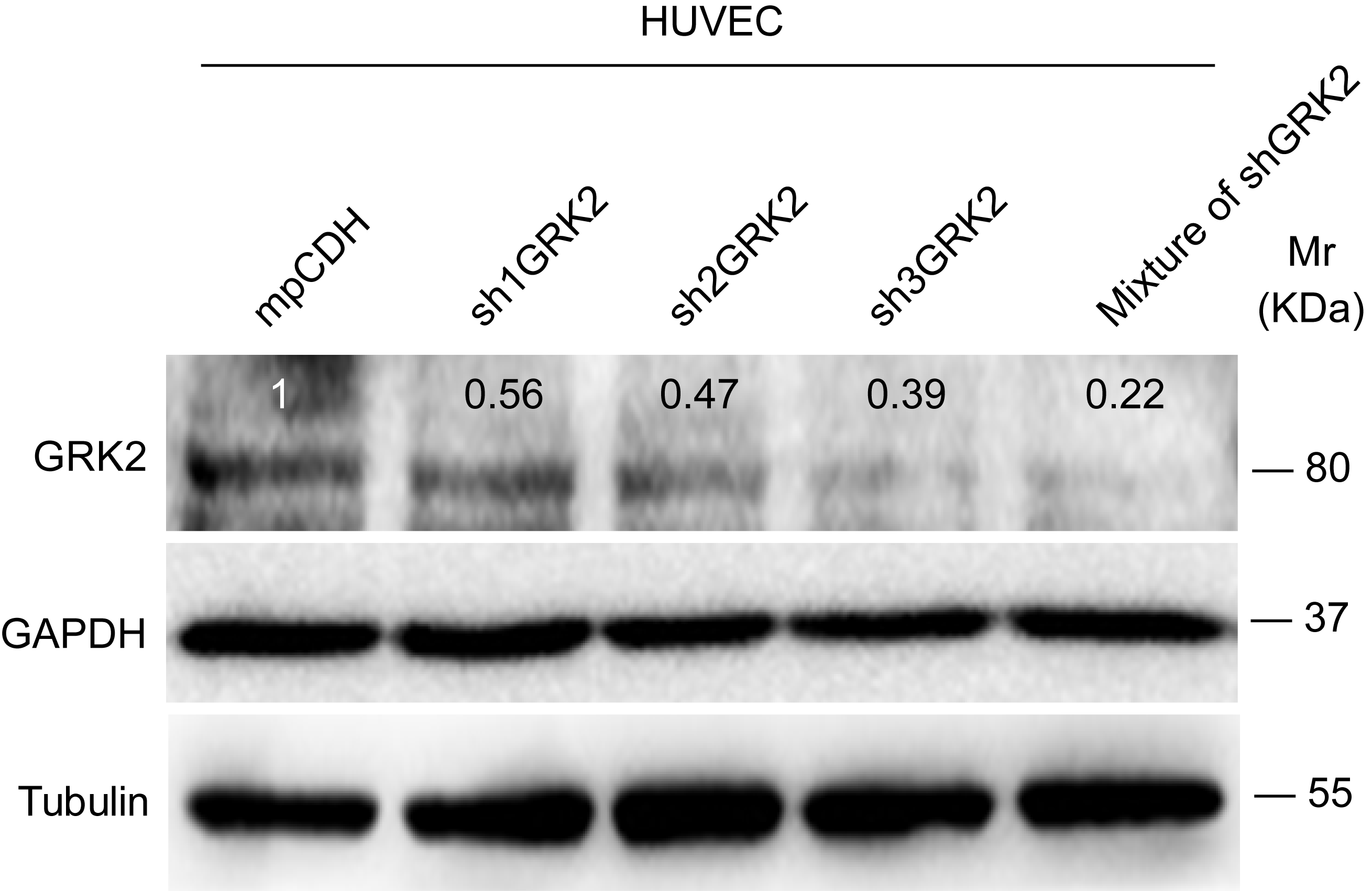

Supplement: S3 Fig — Western blotting was performed in HUVEC transduced with lentivirus-mediated No.1 (sh1GRK2), No. 2 (sh2GRK2), No. 3 (sh3GRK2), and a mixture of No. 1, 2, and 3 together (shGRK2) of short hairpin RNAs targeting GRK2 or the control (mpCDH) with the indicated antibodies. (TIF) [file ppat.1005171.s005.tif]

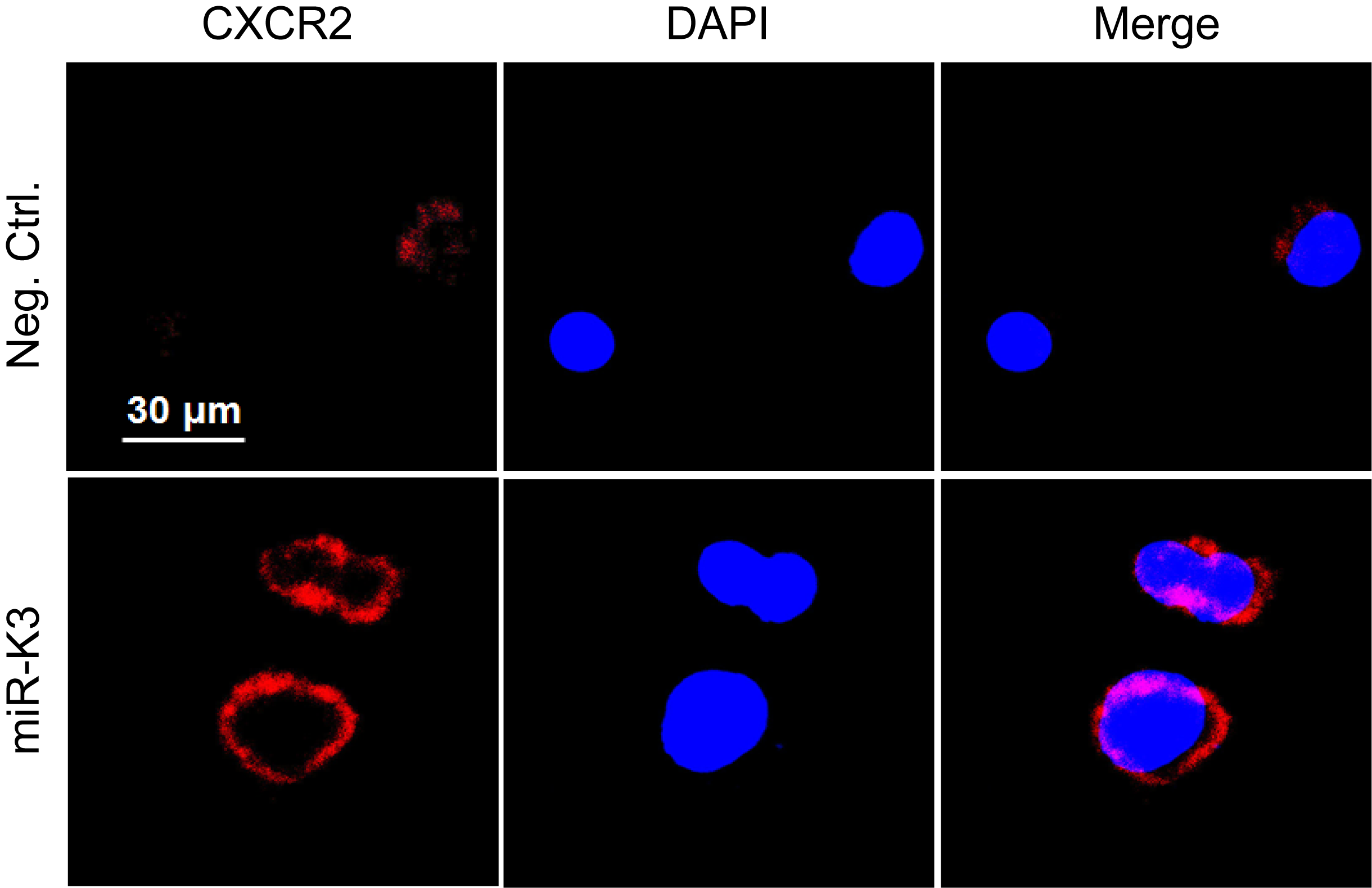

Supplement: S4 Fig — Confocal microscopy of HUVEC transfected by a mimic of miR-K3 (miR-K3) or a negative control nucleotide of miRNA (Neg. Ctrl.), then stained for red fluorescence protein (refers to CXCR2; red). 4’, 6’-diamidino-2-phenylindole (DAPI) (blue) stains nuclei. (TIF) [file ppat.1005171.s006.tif]

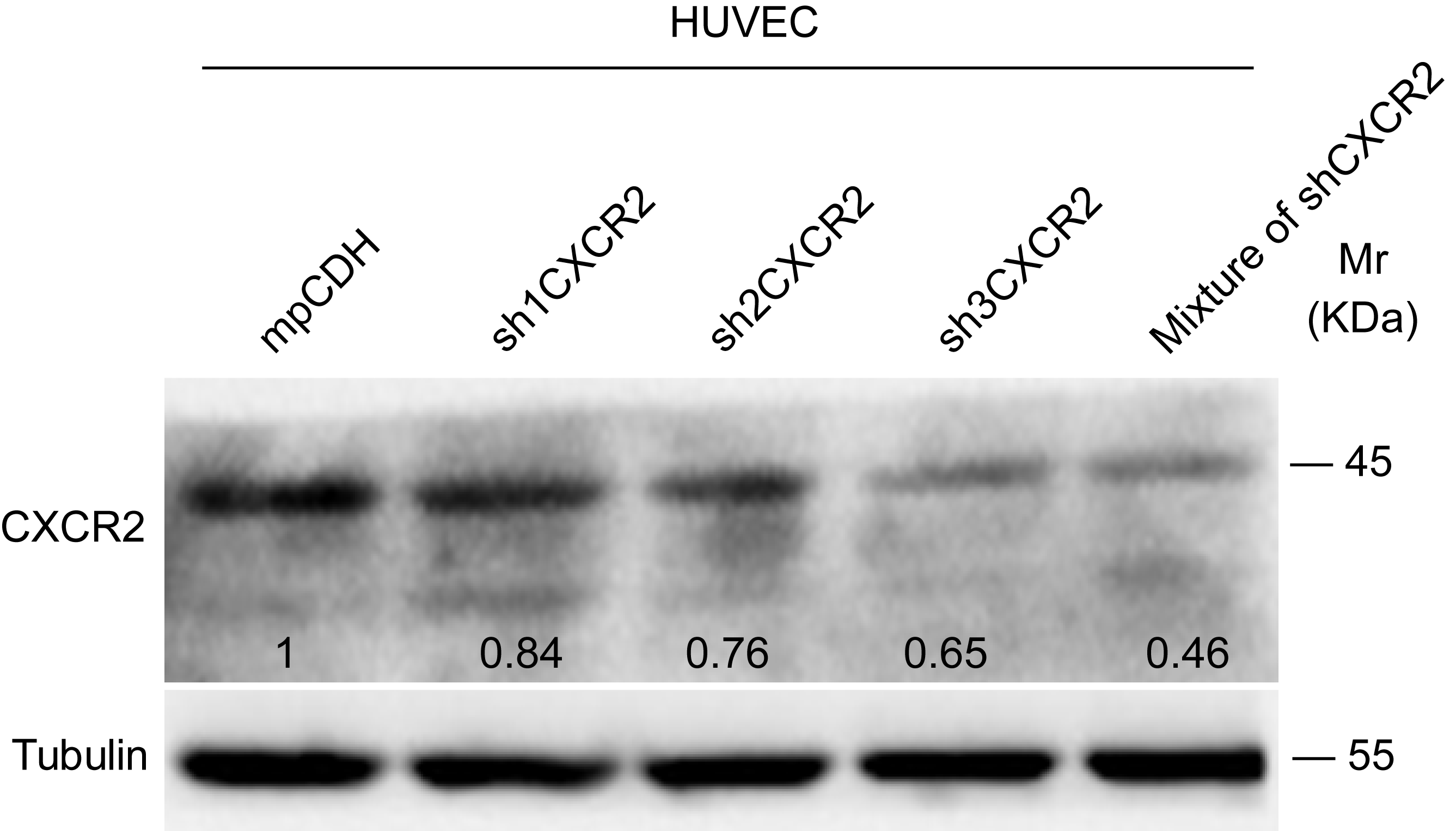

Supplement: S5 Fig — Western blotting was performed in HUVEC transduced with lentivirus-mediated No.1 (sh1CXCR2), No. 2 (sh2CXCR2), No. 3 (sh3CXCR2), and a mixture of No. 1, 2, and 3 together (shCXCR2) of short hairpin RNAs targeting CXCR2 or the control (mpCDH) with the indicated antibodies. (TIF) [file ppat.1005171.s007.tif]

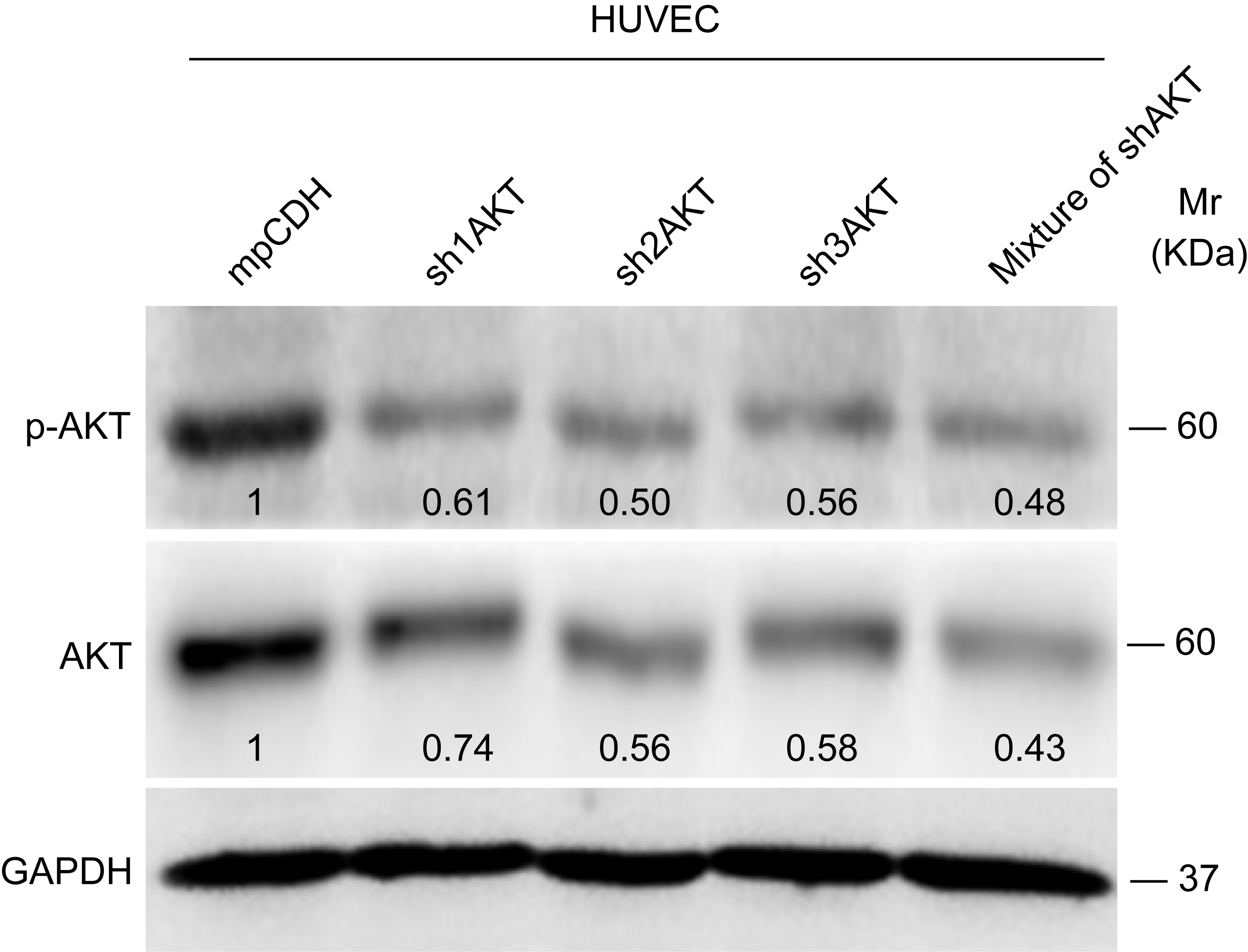

Supplement: S6 Fig — Western blotting was performed in HUVEC transduced with lentivirus-mediated No.1 (sh1AKT), No. 2 (sh2AKT), No. 3 (sh3AKT), and a mixture of No. 1, 2, and 3 together (shAKT) of short hairpin RNAs targeting AKT or the control (mpCDH) with the indicated antibodies. (TIF) [file ppat.1005171.s008.tif]

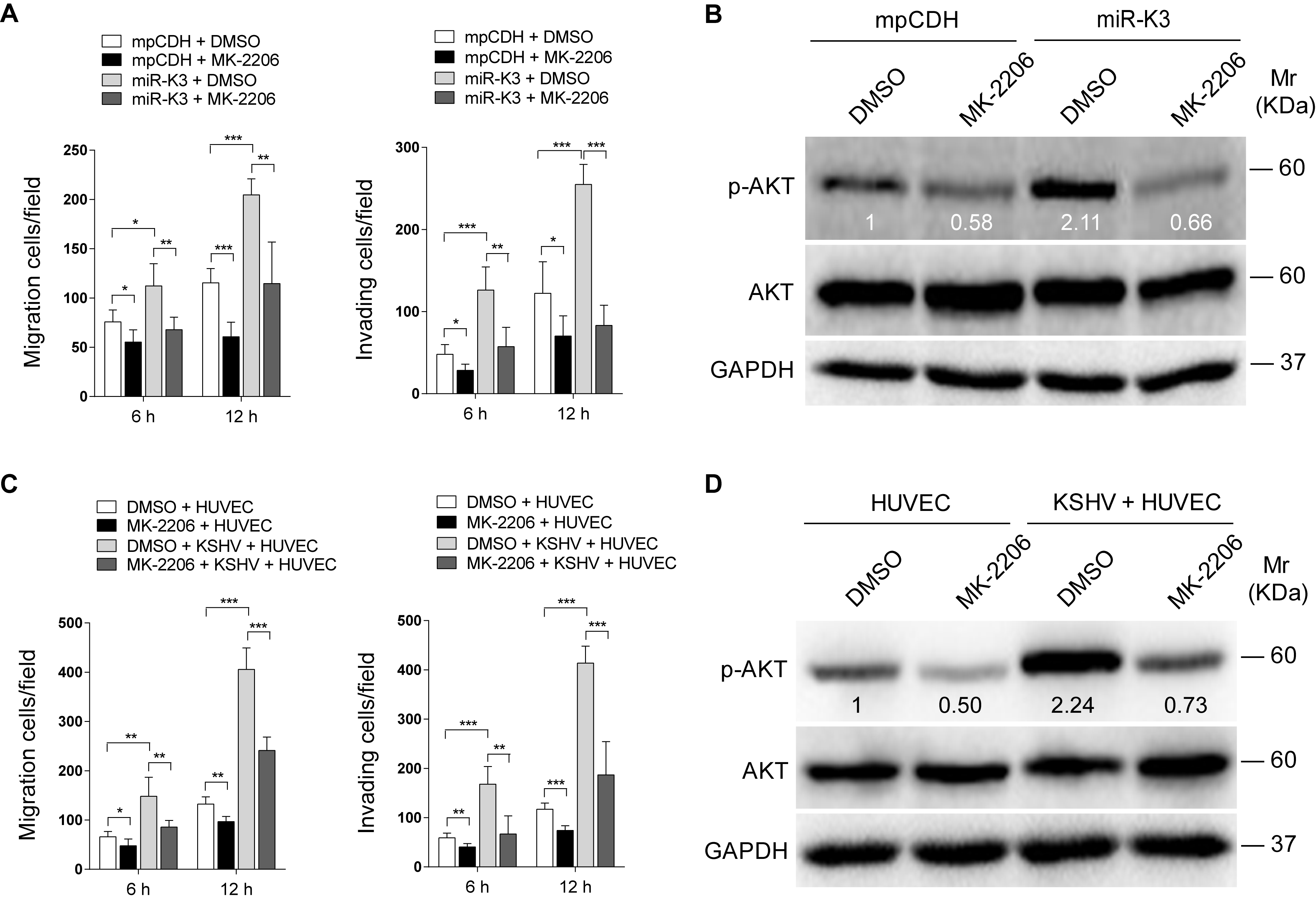

Supplement: S7 Fig — (A). Transwell migration (Left panel) and Matrigel invasion (Right panel) assays for HUVEC which were transduced with lentivirus-mediated empty vector (mpCDH) or miR-K3 (miR-K3) expression and further treated with the AKT inhibitor, MK-2206 (MK-2206) or its control (DMSO). * P < 0.05, ** P < 0.01 and *** P < 0.001 for Student’s t-test. (B). Western blotting analysis of phosphorylated AKT in HUVEC treated as in (A). (C). Transwell migration (Left panel) and Matrigel invasion (Right panel) assays for KSHV-infected HUVEC treated with the AKT inhibitor, MK-2206 (MK-2206) or its control (DMSO). * P < 0.05, ** P < 0.01 and *** P < 0.001 for Student’s t-test. (D). Western blotting analysis of phosphorylated AKT levels in HUVEC treated as in (C). (TIF) [file ppat.1005171.s009.tif]

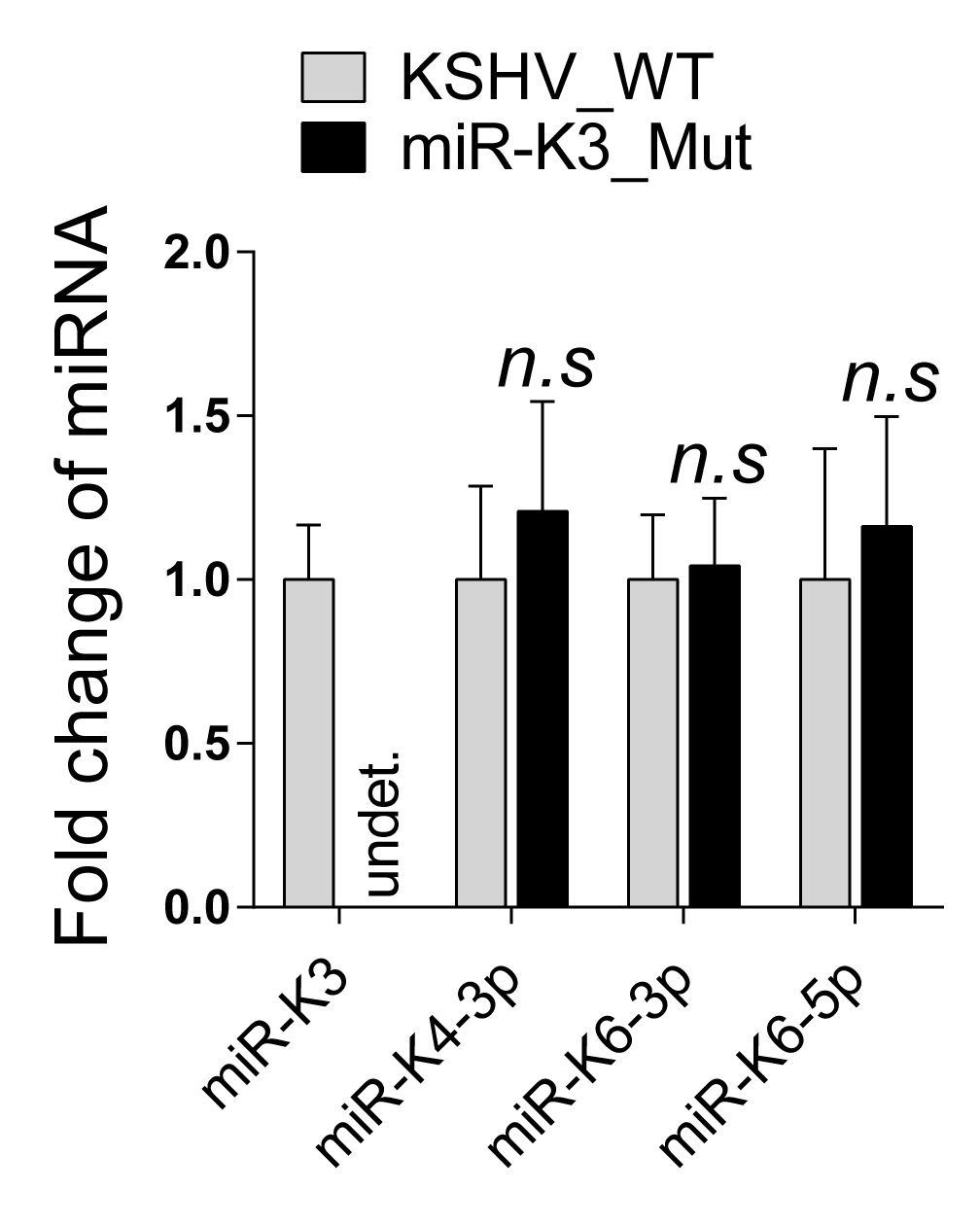

Supplement: S8 Fig — Total RNA was extracted from HUVEC infected with BAC16 KSHV wide type virus (KSHV_WT) or BAC16 KSHV miR-K3 deletion mutant virus (miR-K3_Mut), and levels of KSHV miRNAs miR-K3, -K4-3p, -K6-3p, and -K6-5p were measured by using qPCR. undet, undetermined. n.s., not significant. (TIF) [file ppat.1005171.s010.tif]

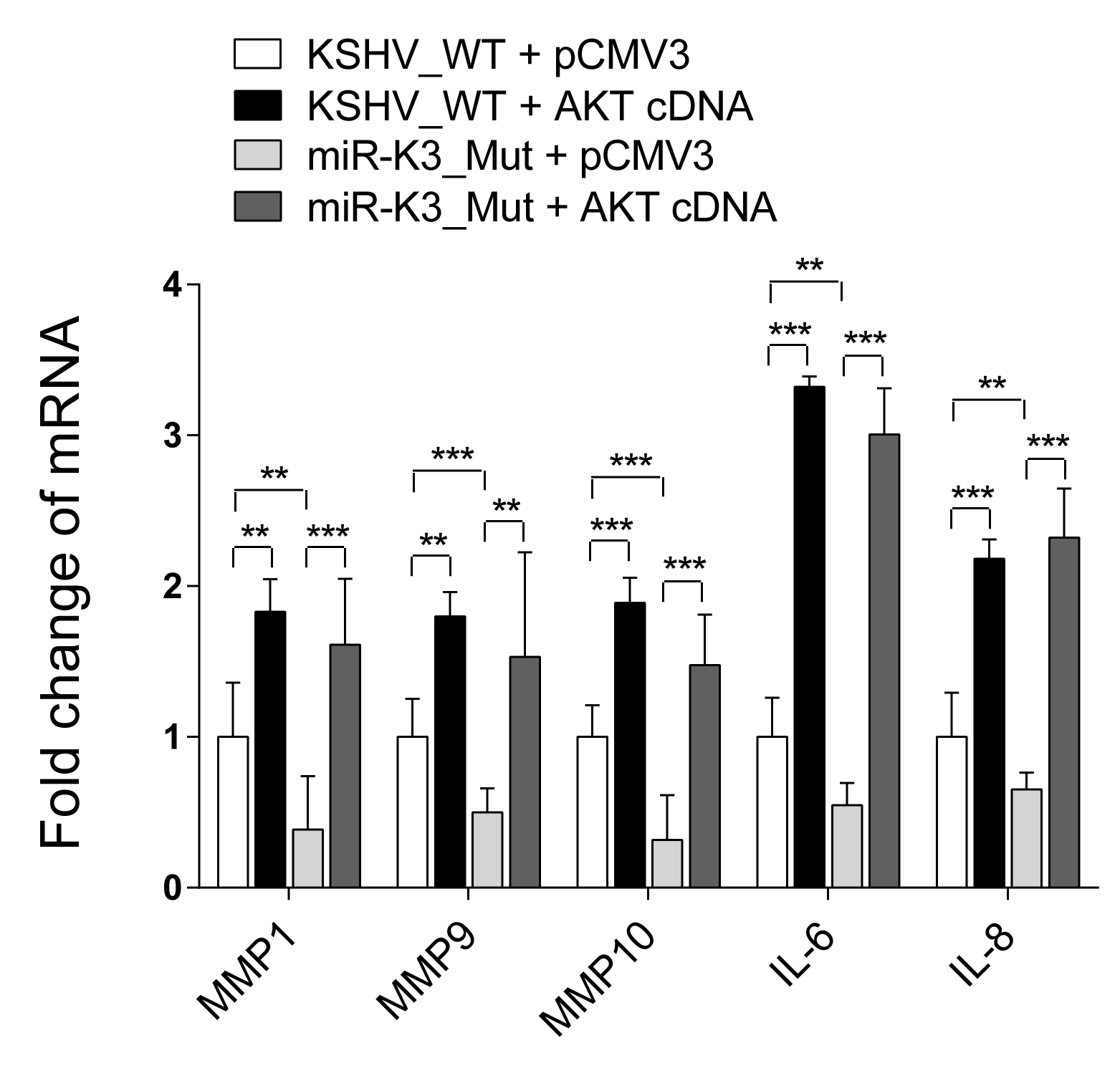

Supplement: S9 Fig — Total RNA was extracted from the BAC16 KSHV wide type virus (KSHV_WT)- or BAC16 KSHV miR-K3 deletion mutant virus (miR-K3_Mut)-infected HUVEC, which were further transfected with pCMV3-HA-AKT construct (AKT cDNA) or its corresponding control pCMV3-C-HA (pCMV). The mRNA expression of MMP1, 9, 10 and IL-6, 8 were determined by qPCR. (TIF) [file ppat.1005171.s011.tif]
